# Spacer acquisition by Type III CRISPR–Cas system during bacteriophage infection of *Thermus thermophilus*

**DOI:** 10.1093/nar/gkaa685

**Published:** 2020-08-21

**Authors:** Daria Artamonova, Karyna Karneyeva, Sofia Medvedeva, Evgeny Klimuk, Matvey Kolesnik, Anna Yasinskaya, Aleksei Samolygo, Konstantin Severinov

**Affiliations:** Center of Life Science, Skolkovo Institute of Science and Technology, Moscow 121205, Russia; Center of Life Science, Skolkovo Institute of Science and Technology, Moscow 121205, Russia; Center of Life Science, Skolkovo Institute of Science and Technology, Moscow 121205, Russia; Center of Life Science, Skolkovo Institute of Science and Technology, Moscow 121205, Russia; Institute of Molecular Genetics, Russian Academy of Sciences, Moscow 123182, Russia; Center of Life Science, Skolkovo Institute of Science and Technology, Moscow 121205, Russia; Center of Life Science, Skolkovo Institute of Science and Technology, Moscow 121205, Russia; Center of Life Science, Skolkovo Institute of Science and Technology, Moscow 121205, Russia; Center of Life Science, Skolkovo Institute of Science and Technology, Moscow 121205, Russia; Institute of Molecular Genetics, Russian Academy of Sciences, Moscow 123182, Russia; Waksman Institute, Rutgers, The State University of New Jersey, NJ 08854 USA

## Abstract

Type III CRISPR–Cas systems provide immunity to foreign DNA by targeting its transcripts. Target recognition activates RNases and DNases that may either destroy foreign DNA directly or elicit collateral damage inducing death of infected cells. While some Type III systems encode a reverse transcriptase to acquire spacers from foreign transcripts, most contain conventional spacer acquisition machinery found in DNA-targeting systems. We studied Type III spacer acquisition in phage-infected *Thermus thermophilus*, a bacterium that lacks either a standalone reverse transcriptase or its fusion to spacer integrase Cas1. Cells with spacers targeting a subset of phage transcripts survived the infection, indicating that Type III immunity does not operate through altruistic suicide. In the absence of selection spacers were acquired from both strands of phage DNA, indicating that no mechanism ensuring acquisition of RNA-targeting spacers exists. Spacers that protect the host from the phage demonstrate a very strong strand bias due to positive selection during infection. Phages that escaped Type III interference accumulated deletions of integral number of codons in an essential gene and much longer deletions in a non-essential gene. This and the fact that Type III immunity can be provided by plasmid-borne mini-arrays open ways for genomic manipulation of *Thermus* phages.

## INTRODUCTION

CRISPR–Cas systems are adaptive heritable immunity systems that provide most prokaryotes (1) and some of their viruses (2,3) an ability to destroy foreign nucleic acids (4). These defense systems can acquire and store fragments of foreign nucleic acid sequences and utilize these fragments as guides to recognize genetic invaders (1). CRISPR–Cas systems comprise CRISPR arrays that consist of two or more identical repeats separated by unique spacers and adjacent clusters of *cas* genes (5). The immune response mediated by CRISPR–Cas systems can be generally divided into three stages (6,7). At the adaptation stage, short fragments of DNA are inserted in the CRISPR array forming a new spacer (1,8,9). A new copy of repeat is also generated at this stage (8–10). At the expression stage, CRISPR arrays are transcribed and short CRISPR RNAs (crRNAs) are produced (4,11). At the interference stage, crRNAs interact with Cas proteins forming effector complexes (effectors) that recognize nucleic acids complementary to crRNA spacer part—protospacers, leading to their cleavage and destruction of the invader (4,12–14).

While the mechanisms of spacer integration are relatively conserved in most of the known CRISPR–Cas systems and rely on the Cas1 integrase (8,15), the mechanisms of crRNA maturation and the structures and activities of effector complexes vary greatly. To date, six CRISPR–Cas systems types and numerous subtypes have been identified based on the structure of the effector complex and the presence of signature Cas proteins (15). In CRISPR–Cas systems belonging to Types II, V and VI the destruction of DNA (Types II and V) or RNA (Type VI) is mediated by effector complexes directly (16–18). Type I effector complexes recruit additional Cas nucleases to destroy the recognized DNA (4). Type IV are degenerate systems that usually lack adaptation modules (15); little is known about their activities (19,20). The Type III CRISPR–Cas systems stand out because of the complexity of their immune response mechanism (21). Type III effector complexes specifically recognize and cleave RNA complementary to crRNA spacer part (13,22,23). Binding to a nascent target RNA activates DNase (24,25) and cyclase domains of the Cas10 subunit of multisubunit Type III effector complexes (26,27). Next, Cas10 nonspecifically cleaves single-stranded DNA (28,29) and produces small cyclic oligonucleotides which act as signaling molecules to activate auxiliary effectors, including non-specific Csm6/Csx1 RNases (26,27,30) belonging to CARF (CRISPR-associated Rossmann fold) family (31) and Can1 and NucC DNases (32,33).

Though its effectors specifically recognize RNA molecules, the Type III immunity protects cells from phages with DNA genomes and interferes with plasmid transformation. This, however, requires that phage or plasmid DNA is transcribed, producing RNA molecules with a protospacer complementary to crRNAs (24,34). It was shown that the efficiency of Type III-mediated immune response depends on the level of protospacer transcription (35). It, therefore, follows that mechanisms allowing to preferentially acquire spacers targeting abundant transcripts should be beneficial for the cell. Some Type III CRISPR–Cas loci encode Cas1 proteins fused to reverse transcriptase (RT) domains suggesting that these systems could acquire spacers directly from RNA molecules (36–38). Indeed, the ability of RT-Cas1 containing adaptation modules to acquire spacers from transcripts was validated experimentally at conditions of adaptation proteins overexpression in either homologous or heterologous hosts. The deletion of the RT domain abolished specific acquisition of RNA-derived spacers (39,40). Most Type III CRISPR–Cas loci lack genes coding for proteins with RT domains (39) implying that they either utilize spacer adaptation mechanisms that are indifferent to protospacer transcription or employ other, yet unexplored, mechanisms to acquire spacers from transcriptionally active sites. At this moment, RT-independent acquisition of spacers for a Type III CRISPR–Cas system was reported only in *Pyrococcus furiosus*. The genome of this archaeon carries three CRISPR–Cas systems of the I-A, I-G, and III-B subtypes and only one locus encoding genes of the adaptation machinery. It was shown that crRNAs from any one of the multiple *P. furiosus* CRISPR arrays, each containing identical repeats, can associate with effector complexes belonging to either of the three subtypes (41). Since in this experimental system there are no CRISPR arrays and adaptation modules that are exclusively employed by the Type III CRISPR–Cas system, specific traits intrinsic to spacer acquisition mechanisms of Type III systems can not be investigated. In this work, we report robust adaptation by the Type III (subtypes III-A and III-B) CRISPR–Cas systems in phage-infected hyperthermophilic gram-negative bacterium *Thermus thermophilus* that lacks either RT-Cas1 fusions or standalone RT genes. We analyze the acquired spacers and their ability to protect the host from the phage and describe a simple system for selection of phages with lesions in specific loci that arise due to Type III targeting.

## MATERIALS AND METHODS

### Strains and cultivation conditions

*Thermus thermophilus* HB27c (42,43) was constructed in the lab of Professor J. Berenguer by the introduction of ∼30 kb insertion carrying a nitrate respiration conjugative element from a megaplasmid of *T. thermophilus* NAR1 strain (GenBank: LR027520.1) into the megaplasmid of *T. thermophilus* HB27 (GenBank: AE017222.1). Cells were cultivated in Innova 40 (New Brunswick Scientific) orbital shaker in the TBM medium (0.8% w/v tryptone, 0.4% w/v NaCl, 0.2% w/v yeast extract in ‘Vittel’ mineral water) supplemented with 0.5 mM MgSO_4_ and 0.5 mM CaCl_2_ at 70°C, 170 rpm. For cultivation on plates, 2% (w/v) agar and, where appropriate, 0.7% (w/v) top agar was added. Plates were incubated at 67°C in RedLine RI 53 (Binder) incubator.

*Escherichia coli* DH5α (F^−^ Φ80*lac*ZΔM15 Δ(*lac*ZYA-*arg*F) U169 *rec*A1 *end*A1 *hsd*R17(r_k_^−^, m_k_^+^) *pho*A *sup*E44 *thi*-1 *gyr*A96 *rel*A1 λ^−^) was used for molecular cloning. Cells were cultivated in LB medium at 37°C, agar was added up to 1.5% (w/v) for growth on plates.

Bacteriophages phiFa (MH673672.2) and phiKo (MH673671.3) were isolated in our laboratory (44). To prepare phage lysates, 2 ml of fresh *T. thermophilus* HB27c cell culture (OD_600_ ∼ 0.2) was infected with a single phiFa or phiKo plaque and cultivated for 2–3 h at standard *T. thermophilus* growth conditions (above). Cell debris was removed by centrifugation (10 000 g) and the supernatant was transferred into 10–50 ml of fresh bacterial culture (OD_600_ ∼ 0.2). After 2–3 h of cultivation, the infected culture was centrifuged, the supernatant was collected and stored at 4°C.

### Genomic DNA extraction

Overnight *T. thermophilus* HB27c culture was diluted 1:50 with fresh TBM medium and cells were grown until OD_600_ ∼ 1.0. 5 ml of culture was centrifuged for 10 min at 5000 g. The cell pellet was resuspended in 2.5 ml of Lysis buffer (10 mM Tris–HCl, pH 8; 1 mM EDTA; 0.6% SDS; 20 μg/ml of Proteinase K) and incubated at 60°C for 1 h. Next, triple extraction with 2.5 ml of phenol (pH 8): chloroform: isoamyl alcohol (25:24:1) was performed. The aqueous phase was next extracted twice with an equal volume of chloroform: isoamyl alcohol (24:1). Nucleic acids from aqueous phase were ethanol precipitated in the presence of 0.5 M NaCl, 50 μg/ml glycogen. The pellet was washed with 70% cold ethanol, dried at room temperature, and resuspended in 100 μl TE buffer (10 mM Tris–HCl, pH 8; 1 mM EDTA). 100 μg/ml RNase A was added and the solution was incubated at 37°C for 30 min. This was followed by phenol/chloroform extraction and ethanol precipitation as described above.

### Whole-genome sequencing, genome assembly, and annotation

∼10 μg of total *T. thermophilus* HB27c DNA was used for sequencing on Illumina MiSeq and Oxford Nanopore platforms. 633 368 of 2 × 250 bp pair-end reads were obtained by Illumina sequencing, 64 888 reads were obtained by Oxford Nanopore. Hybrid assembly was performed using SPAdes 3.10.1 (45,46) with default parameters and MismatchCorrector tool. The obtained sequences were annotated using Prokka pipeline (47). The amino acid sequences of ORFs predicted in the annotation of *T. thermophilus* HB27 (48) were used for the annotation by sequence similarity (prokka –protein parameter), the HMM database of prokka was supplemented with Pfam-A profiles v. 32.0 (49).

### CRISPR adaptation assay

A typical experiment lasted three days. An overnight culture of *T. thermophilus* HB27c was diluted 1:50 with TBM medium, grown until OD_600_ reached 0.2 and infected with phiFa or phiKo (MOI ∼ 0.0001). After every 24 h, an aliquot of culture was diluted with a fresh medium (1:500) and growth was continued. CRISPR arrays expansion was detected by PCR with Taq DNA polymerase from a lab-made stock. 1 μl cell culture aliquot was combined with 20 μl of PCR mixture. Oligonucleotides (Evrogen) annealing to the leader and to the leader-proximal spacer of each array were used as forward and reverse primers (Supplementary Table S1), correspondingly. Amplification reactions were conducted under the following conditions: 95°C for 3 min, [95°C for 30 s, 59°C for 20 s, 72°C for 30 s] × 27, 72°C for 3 min.

For isolation of individual clones with expanded arrays, aliquots of cultures were plated on agar medium and each of eleven arrays were checked by PCR in bacterial colonies that appeared after overnight growth. Sequences of acquired spacers were determined by Sanger sequencing.

### Bioinformatics analysis of Cas1 sequences

Amino acid sequences of four Cas1 proteins from *T. thermophilus* HB27c (genes HB27c_P00244, HB27c_P00263, HB27c_C01208 and HB27c_P00143) were aligned with homologous Cas1 sequences from *T. thermophilus* HB8 (genes TTHB145, TTHB193 and TTHB224) and *T. thermophilus* TTHNAR1 (genes TTHNP4_00086 and TTHNP4_00403) with Jalview 2.10.5 (50) using the TcoffeeWS algorithm and the average distance tree was calculated using BLOSUM62. Secondary structures of Cas1 proteins were predicted with the PROMALS3D tool (51).

### Construction of Δ*cas1^1^*, Δ*cas1^4^* and Δ*cas1^1^*Δ*cas1^4^* strains

The Δ*cas1^1^* and Δ*cas1^4^*strains were constructed by replacement of entire *T. thermophilus* HB27c *cas1^1^*or a part of *cas1^4^* (230 codons at the beginning of the gene) with a thermostable hygromycin resistance marker via natural homologous recombination (52). The Δ*cas1^1^*Δ*cas1^4^*double mutant was constructed from the Δ*cas1^1^* strain by replacement of the part of *cas1^4^*mentioned above with a bleomycin resistance marker.

To construct pT7_Δ*cas1^1^_hygR*, pT7_Δ*cas1^4^_hygR* and pT7_Δ*cas1^4^_bleoR* recombination plasmids, antibiotic resistance genes were amplified from plasmids pMH184 ((53); a kind gift of Prof. J. Berenguer) and pWUR112 ((54); a kind gift of Prof. J. van der Oost). The *cas1* genes flanking areas were amplified from *T. thermophilus* HB27c genomic DNA with primers listed in Supplementary Table S1. The pT7blue (Novagene) plasmid was linearized with EcoRV restriction endonuclease (Thermo Scientific) and dephosphorylated with FastAP (Thermo Scientific). Fragments were separated by electrophoresis in 1% agarose gel and extracted with GeneJET Genomic DNA Purification Kit (Thermo Scientific). Different combinations of four DNA fragments (linearized pT7blue, an antibiotic resistance gene, and left and right recombination arms of *cas1* genes) were incubated with Gibson Assembly Mix (New England Biolabs) according to the manufacturer protocol. Chemically competent *E. coli* DH5α cells (55) were transformed with the mixtures and plated on LB-agar plates supplemented with 100 μg/ml ampicillin. After overnight incubation, clones of interest were verified by PCR. Plasmids from selected clones were purified and verified by Sanger sequencing.

*T. thermophilus* cells were transformed with pT7_Δ*cas1^1^_hygR*, pT7_Δ*cas1^4^_hygR* or pT7_Δ*cas1^4^_bleoR* plasmids according to a protocol described by (56) and recombinant clones were selected on an appropriate antibiotic (50 μg/ml hygromycin B or/and 15 μg/ml bleomycin). After 2–3 passages of isolated clones on selective media, the identity of selected clones was confirmed by PCR and sequencing of amplified fragments.

### phiFa DNA purification, analysis of phiFa genome termini, reassembling of the phiFa genome

30 ml of phage lysate was mixed with 10 ml of 20% PEG-8000 and 2.5 M NaCl solution and incubated at 4°C overnight. The mixture was centrifuged at 11 000 g, 4°C for 30 min. Pellet was resuspended in 1 ml of STE buffer (10 mM Tris–HCl, pH 8.0; 1 mM EDTA; 100 mM NaCl) supplemented with 0.6% SDS and 10 μg/ml Proteinase K and incubated at 55°C for 1 h. Next, DNA was purified with phenol: chloroform: isoamyl alcohol and treated with RNase A as described above. PhiFa genomic DNA was sequenced with Oxford Nanopore platform at the Skoltech Genomics Core Facility. The generated reads were analyzed with PhageTerm tool (57) on a Galaxy-based server (https://galaxy.pasteur.fr) with default parameters. To precise the sequence of phiFa genome, a hybrid reassembling of Illumina and Oxford Nanopore reads were performed with SPAdes 3.13.0.

### High-throughput sequencing of acquired spacers

Amplicons corresponding to expanded CRISPR-2 and CRISPR-11 arrays (PCR products ∼200–500 bp in length) were cut from agarose gels and extracted with GeneJET Genomic DNA Purification Kit (Thermo Scientific). Samples from two independent biological replicas for each array were taken for analysis. DNA libraries were prepared and sequenced with Illumina MiSeq device of Skoltech Genomics Core Facility. 2 × 250 bp pair-end reads were obtained. Custom R scripts using ShortRead Bioconductor package (58) were applied for quality filtration and analysis. CRISPR repeat sequences in reads were identified and sequences between them were considered as new spacers. All acquired spacers (up to five in one pair-end read) were pooled together and the joint dataset was analyzed. Extracted spacers were mapped on the phiFa/phiKo genomes and *T. thermophilus* HB27c chromosome and megaplasmid sequences with BLAST+ application. 95% identity threshold was set for mapping. Only uniquely mapped spacers with lengths no <20 bp were considered. Spacer sequences were clustered based on the start position, length, and a strand they map to. Spacers mapped to predicted CRISPR arrays were removed in order to keep only newly acquired spacers. Clusters of spacers with the same start positions and strand were combined before visualization of spacer distribution across the phage genome. Weblogo plots were built using ggseqlogo package (59).

### Bacterial growth curves

TBM medium was inoculated with single colonies and cultures were allowed to grow until OD_600_ reached 0.2–0.3. Phage was added to the MOI of 5 and growth was allowed to continue. OD_600_ measurements were carried out with OD_600_ DiluPhotometer (Implen) every 20 min during the first hour, and then at 2 and 3 h post-infection. Growth of uninfected cultures was monitored in parallel.

### Construction of plasmids bearing CRISPR mini-arrays

Complementary oligonucleotides that consisted of a spacer sequence flanked by Type III repeats (Supplementary Table S1) were annealed and cloned between the SalI and HindIII sites of the *E. coli–T. thermophilus* shuttle vector pMK18 which was modified by insertion of a transcription terminator downstream of the multiple cloning site (35). To construct plasmids bearing CRISPR mini-arrays and protospacers matching spacer sequences, complementary protospacer oligonucleotides (Supplementary Table S1) were annealed and cloned into the PvuI site of pMK18-based plasmids bearing corresponding mini-arrays. *E. coli* clones carrying recombinant plasmids were selected on LB medium supplied with agar and 50 μg/ml kanamycin and the presence of required inserts was confirmed by PCR and verified by Sanger sequencing.

### Phage interference assay

Serial 10x dilutions of phage stock lysates were spotted on double-layer TBM agar plates freshly seeded with lawns of various *T. thermophilus* HB27c strains. When testing strains bearing pMK18-based plasmids with artificial Type III mini-arrays, the plates were supplemented with 30 μg/ml kanamycin in the bottom layer. After overnight incubation interference efficiency was estimated.

### Isolation of escaper phages

Fa_E42 and Fa_E44 *T. thermophilus* HB27c cell cultures (OD_600_ = 0.2) were infected with phiFa (MOI = 5), incubated overnight and supernatants were collected. Aliquots of serial dilutions of supernatants were plated on corresponding lawns of cells. Individual plaques were used to prepare escaper phage stocks.

### Analysis of escaper diversity

The supernatants obtained from overnight cultures of infected Fa_E42 and Fa_E44 cells were used as templates for PCR. The regions of phage DNA around targeted protospacers were amplified with primer pairs listed in Supplementary Table S1. Following the purification of amplified products, samples were subjected to MiSeq Illumina sequencing. 2 × 150 paired reads were trimmed from low-quality sequences and merged with PEAR (60). Fastq files were transformed into fasta format and identical sequences were clustered using USEARCH (61). Sequences, which did not contain both primers at the ends were removed from the analysis. Distributions of deletion sizes were calculated with R script and visualized using Plotly package (62).

### Full genome sequencing of escaper phages

DNA was extracted from four randomly picked escapers and a control wild-type phiFa phage lysates using the protocol described previously. Samples were sequenced with Illumina Miseq platform, 2 × 75 paired-end reads were obtained. Reads were mapped to the reference sequence of phiFa with bowtie2 (63). Coverage was calculated with bedtools (64) and analyzed in IGV browser (65).

### Determination of temporal classes of phiKo genes

*T. thermophilus* HB27c culture (OD_600_ = 0.2) was infected with phiKo (MOI = 10). Aliquots of the culture (15 ml) were collected before infection (as a control) and 10, 30, 50 and 70 min post-infection. Total RNA was extracted from the cells following the standard protocol with TRI Reagent (Sigma-Aldrich) and treated with RNase-free DNase I (Thermo Fisher Scientific). RNA sequencing was carried out at an Illumina platform using the resources of the Skoltech Genomics Core Facility. The raw reads were subjected to quality filtering and adaptor trimming using Trimmomatic v0.3833 (66) with the following parameters: SE-phred33 Illuminaclip:TruSeq3-se:2:30:10 leading:3 trailing:3 slidingwindow:4:15 minlen:36. The quality before and after processing was examined using FastQC tool. Processed reads were mapped to the reference sequences (phiKo genome (GenBank ID: MH673671.3) and the *T. thermophilus* HB27c chromosome and megaplasmid (BioProject ID PRJNA631468) using bowtie2 v2.4.1 (63) with default settings. The quantification of reads by phage genes was performed using featureCounts function from the Rsubread package v2.2.2 (67) in a strandless mode and allowed multiple overlapping of reads with features; other parameters were set to default. TPM (transcripts per million) values were calculated with normalization on a total number of counted reads. These TPM values were used to create a heat map.

Each phiKo gene was assigned to one of three temporal classes—early, middle or late—according to its transcript abundance within a certain period post infection. The dynamics of transcript abundance was quantified with the help of a Log-Fold Change parameter (LogFC) that was calculated as follows: LogFC_XvsY = log10A(Y) – log10A(X), where A(X) and A(Y) are normalized transcript abundances of the gene at time points X and Y post infection. The maximum values of transcript abundances of the Early class genes was expected to be within the first 30 min post infection, so their LogFC values obeyed the following criterion: LogFC_30vs50 < 0. We expect the transcript abundances of middle class genes are increased during 30 min post-infection and decreased after 50 min post-infection: logFC_30vs50 > 0 and logFC_50vs70 < 0. The transcript abundances of the late class genes are increased after 50 min post-infection: logFC_50vs70 > 0. Data obtained from two biological replicas were used for analysis.

## RESULTS

### High-throughput sequencing reveals an extra *cas1* gene and a Type III CRISPR array in *Thermus thermophilus* HB27с genome

**Figure 1.**
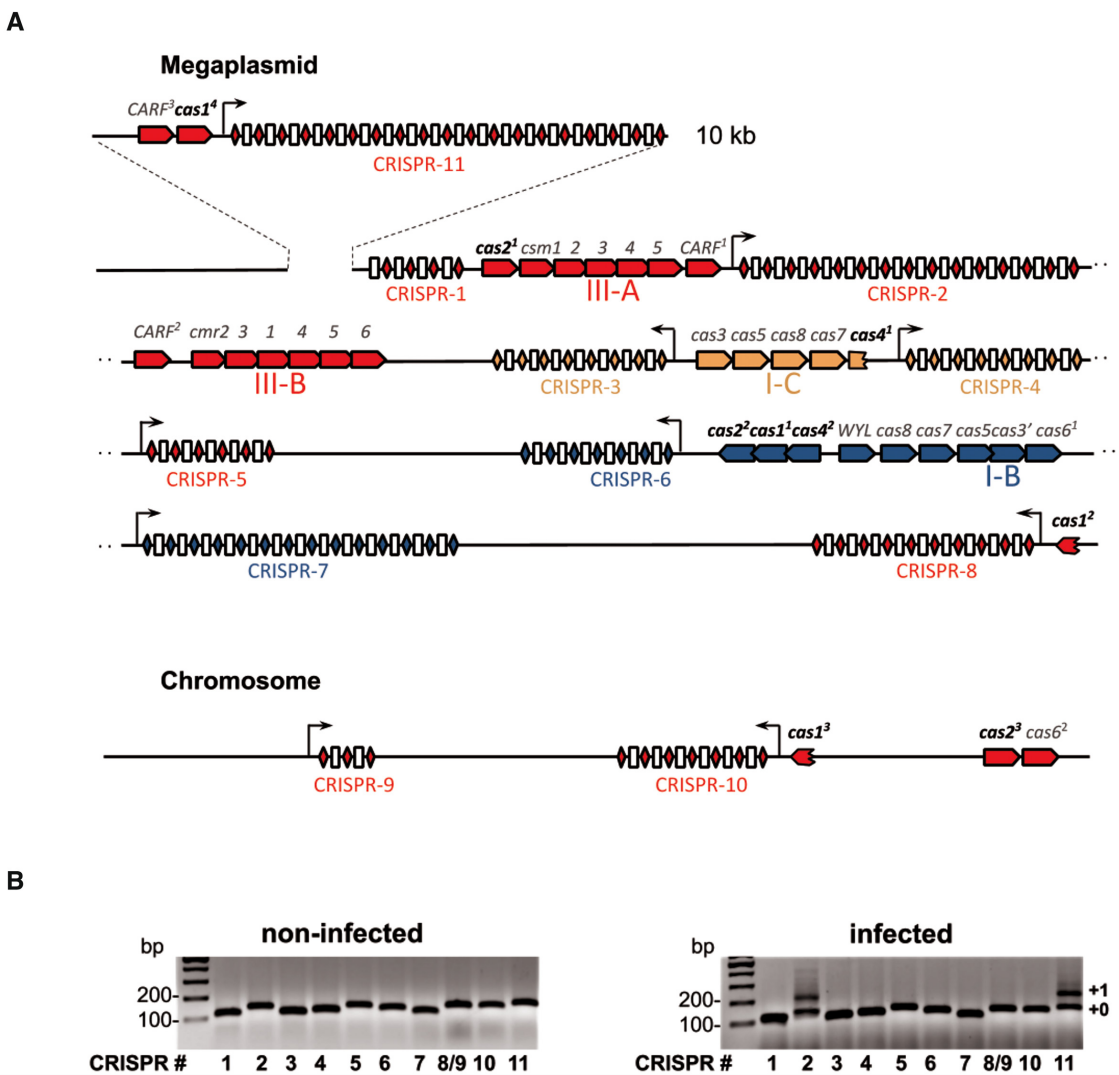
The CRISPR–Cas systems of *T. thermophilus* HB27c and spacer acquisition in phiFa phage infected culture. (**A**) CRISPR–Cas loci located on the *Thermus thermophilus* HB27c megaplasmid and chromosome are shown. CRISPR arrays are numbered and the direction of their transcription is marked with arrows. CRISPR repeats are indicated as color-coded rhombi (blue – I-B, orange – I-C, red – III-A and III-B subtypes), spacers are shown as white rectangles. The *cas* genes are shown as pentagons and are colored according to their subtype. A 10 kb insertion detected in the HB27c megaplasmid and carrying the CRISPR-11 array and two CRISPR-related genes is shown on the top of the scheme. (**B**) Expansion of *T. thermophilus* HB27c CRISPR arrays in uninfected cultures and in cultures infected with bacteriophage phiFa was followed by PCR with primers annealing to leaders and leader-proximal spacers of indicated arrays. Amplification products were separated by agarose gel electrophoresis and visualized by ethidium bromide staining. Lane numbering coincides with CRISPR arrays numbers in panel А (the CRISPR-8 and CRISPR-9 arrays have identical leader-proximal spacers and highly similar leaders and were amplified with the same primer pair). ‘+0’ and ‘+1’ indicate, correspondingly, amplicons of non-expanded arrays and arrays expanded by one spacer-repeat unit.

The *T. thermophilus* strain HB27с, a derivative of *T. thermophilus* HB27 (GenBank: AE017221.1 and AE017222.1) containing a nitrate respiration conjugative element (NCE) from *T. thermophilus* TTHNAR1 (GenBank: LR027517.1–LR027520.1), was used as a model organism in this study. The HB27с genome (42,43) consists of a circular chromosome and a megaplasmid and, based on its pedigree, should encode I-B, I-C, III-A and III-B subtype CRISPR–Cas systems and ten CRISPR arrays with three different types of repeats (Figure 1A, Supplementary Table S2). Two arrays are assigned to the subtype I-B system, two - to subtype I-C, and the remaining six are shared by the III-A and III-B subtypes (44). Given the reported variability of *T. thermophilus* strains (68), we determined the full genomic sequence of the isolate maintained in our laboratory. The results revealed high similarity to the published HB27 genome and the presence of the NCE insertion from TTHNAR1, as expected. Several deletions, insertions, and mismatches compared to the reference genome were observed. The only difference related to CRISPR–Cas systems—an insertion of ∼10 kb fragment that is absent from published HB27 and TTHNAR1 genomes—was found within the megaplasmid sequence of our HB27с strain (Figure 1A). This fragment contains seven open reading frames (ORFs), of which two encode CRISPR-associated proteins: a *cas1* gene (labeled *cas1^4^* on Figure 1A) and a gene belonging to CARF family (31) (labeled *CARF^3^* on Figure 1A). A Type III CRISPR array with 18 spacers that we will refer to as CRISPR-11 was also located in the ∼10 kb fragment. 17 spacers of CRISPR-11 are unique and have no matches with sequences in the public database. One spacer partially matches a Type III CRISPR array spacer from *T. thermophilus* HB8. The HB27c spacer is four nucleotides shorter and offset by 4 bases compared to the one in HB8 and thus must have been acquired independently from an unknown mobile genetic element. Coordinates of all CRISPR-associated genes of *T. thermophilus* HB27c shown in Figure 1A are listed in Supplementary Table S3.

### Some *T. thermophilus* HB27c Type III arrays acquire spacers during bacteriophage infection

The HB27c culture was infected with lytic bacteriophage phiFa (44) and expansion of each of the eleven CRISPR arrays was monitored by PCR over the course of several days. Experiment was performed at low initial multiplicity of infection (MOI) since high MOI was reported to promote the appearance of resistant clones that arise due to defects in phage absorption (69). Uninfected cells were used as control. Expansion of Type III CRISPR-2 and CRISPR-11 arrays was detected in infected but not in uninfected cultures (Figure 1B). We routinely observed highly variable levels of adaptation in cultures infected in parallel (Supplementary Figure S1). Moreover, the extent of adaptation by CRISPR-2 and CRISPR-11 arrays in the same culture also varied in parallel cultures.

*T. thermophilus* HB27c encodes two full-sized Cas1 proteins, Cas1^1^ and Cas1^4^ (325 and 315 amino acids, respectively), and two truncated versions, Cas1^2^ and Cas1^3^ (74 and 87 amino acids, respectively) (Figure 1A, Supplementary Table S3). Analysis of Cas1^2^ and Cas1^3^ sequences reveals that they correspond to C-terminal portions of full-sized Cas1 and cannot be functional in adaptation since they do not have conserved essential amino acids needed to coordinate catalytic divalent metal ions (Supplementary Figure S2A). The HB27c gene encoding Cas1^1^ is located in the subtype I-B CRISPR–Cas locus (Figure 1A). Highly similar proteins are also encoded in subtype I-B CRISPR–Cas loci of *T. thermophilus* HB8 and TTHNAR1 (Supplementary Figure S2B). In contrast, the Cas1^4^ protein is 97% identical to Cas1_TTHB145 encoded by a subtype III-A *cas* locus of *T. thermophilus* HB8 (Supplementary Figure S2A) and thus may be responsible for spacer acquisition by Type III CRISPR-2 and CRISPR-11 arrays. We constructed HB27c derivatives lacking *cas1^1^*, *cas1^4^*, or both genes. During phage infection, robust adaptation by CRISPR-2 and CRISPR-11 arrays occurred in parental and Δ*cas1^1^* strains but not in Δ*cas1^4^* or the double mutant (Supplementary Figure S2C). We conclude that the product of *cas1^4^* is responsible for Type III adaptation.

### Most newly acquired spacers originate from the early region of phage DNA

Amplicons corresponding to expanded CRISPR-2 and CRISPR-11 arrays were purified and subjected to high-throughput sequencing. Newly acquired spacers varied in length from 35 to 42 bp (Supplementary Figure S3A), which matches the naturally observed variation of Type III array spacers lengths in environmental *Thermus* communities (44) and lengths of spacers in *T. thermophilus* HB27c Type III CRISPR arrays (Supplementary Figure S3A). Spacer sequences were mapped on the chromosome, the megaplasmid, and the phiFa genome. The Jaccard similarity (number of shared spacers divided by the number of unique spacers in combined replicas) for numbers of spacers with identical sequences found in different biological replicas or in different arrays from the same sample was less than 2%, (Supplementary Figure S3B), indicating that diversity of acquired spacers is strongly undersampled and there is no strong preference for acquisition of specific spacers. No nucleotide preferences in protospacers or protospacer adjacent sequences were detected (Supplementary Figure S3C), an expected result, since Type III CRISPR–Cas systems do not require a PAM for target recognition (70).

**Figure 2.**
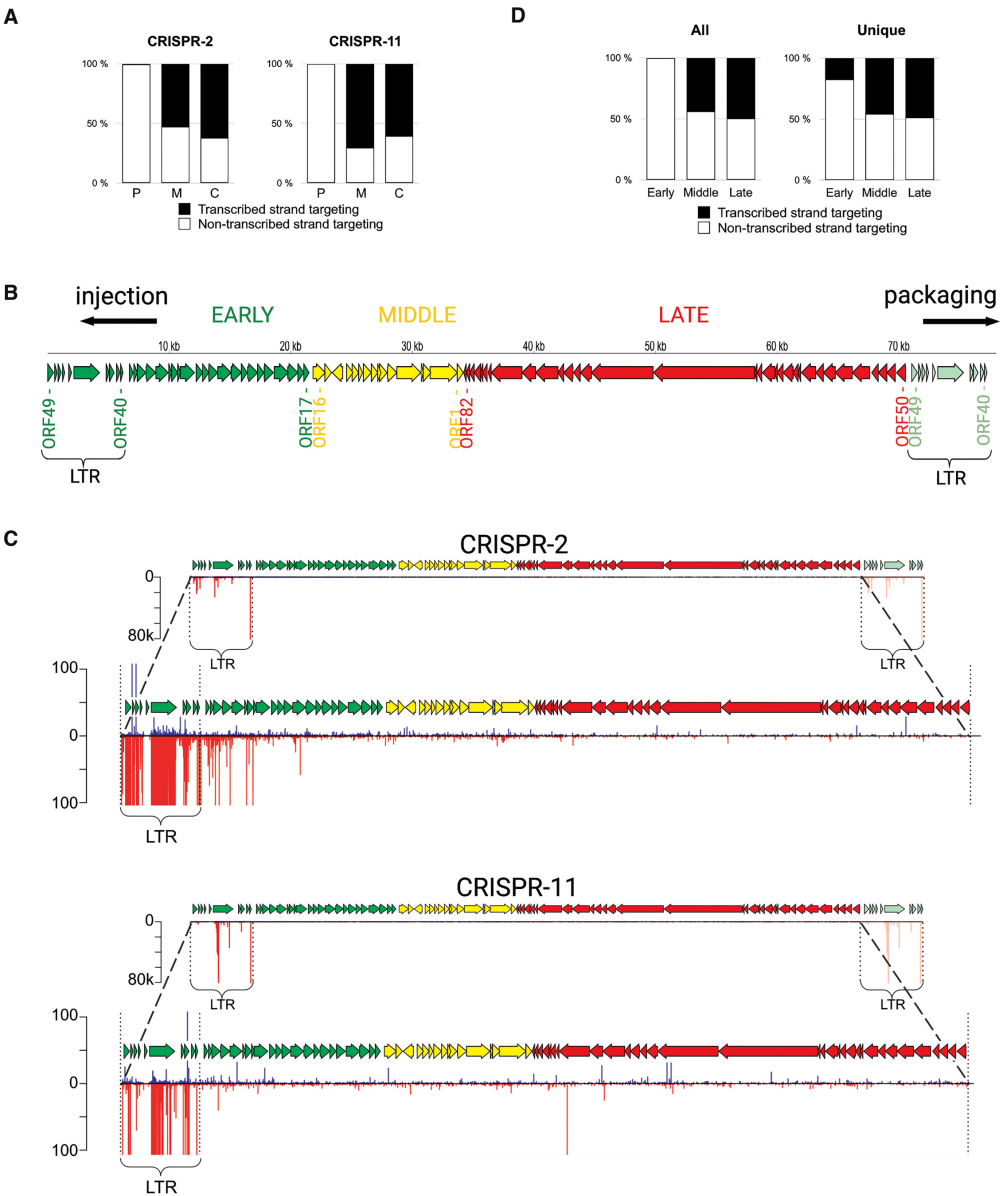
Analysis of spacers acquired by Type III arrays in phiFa infected cells. (**A**) Orientation of spacers acquired by the CRISPR-2 and CRISPR-11 arrays. The fraction of spacers parallel to the orientation of genes they are derived from (targeting the transcribed strand) is shown in black, in antiparallel orientation (targeting the non-transcribed strand) – in white. ‘C’ – spacers acquired from the chromosome, ‘M’ – from the megaplasmid, ‘P’ – from the phiFa genome. (**B**) A revised scheme of phage phiFa genome. Phage genes are shown as colored arrows whose directions match the direction of transcription: green - early genes, yellow - middle, red - late genes. The LTRs and likely directions of phage DNA injection during infection and packaging into virions are shown. (**C**) Mapping of spacers acquired by CRISPR-2 and CRISPR-11 arrays on the phiFa genome. Spacers whose sequences match the ‘top’ strand of phage DNA (5′-3′ direction) are shown as blue bars, those matching the ‘bottom’ strand – as red bars. The height of a bar reflects the quantity of reads corresponding to a particular spacer. The lower views for each array allow one to see minor spacers (scales indicating the number of reads are indicated on the left). (**D**) Orientation of phage-originated spacers acquired from phiFa genome regions encoding different temporal classes of genes (early, middle, late). Spacers extracted from reads corresponding to CRISPR-2 and CRISPR-11 arrays were combined for this analysis. Results for all spacers and unique spacers are shown separately. The fractions of spacers targeting the transcribed strands are shown in black, non-transcribed – in white.

The vast majority (>95%) of spacers were acquired from phage DNA (Supplementary Figure S3D). In contrast to host-originated spacers, which mapped to either transcribed or non-transcribed strands of ORFs, almost all phage-originated spacers were complementary to phage transcripts, Figure 2A).

Most frequent phage-derived spacers mapped to a ‘hot’ region corresponding to early genes *40*–*49*. A similar pattern was previously reported in the course of our studies of Type I-E adaptation in *E. coli* infected with bacteriophage T5, where spacers were exclusively acquired from a small region of phage DNA (71). In the T5 genome, this region (termed ‘pre-early’) (72), forms long terminal repeats (LTRs) that are found at both ends of the genome (73) and arise during phage DNA replication and its packaging in the virions (74). Unlike the case of T5, the region of phiFa from which spacers are acquired is located in the middle of the published genome sequence (44) (Supplementary Figure S4A). We re-sequenced phiFa genomic DNA using the long-read Oxford Nanopore platform. Analysis with PhageTerm tool (57) allowed us to redetermine the termini of the phiFa genome based on (i) the prevalence of reads with defined ends that must correspond to genome termini and (ii) increased coverage of reads of the early region indicative of the presence of LTRs (Supplementary Figure S4B, Figure 2B). By analogy with T5 we propose that the phiFa genome is packaged from a concatemeric precursor starting at a previously annotated nucleotide position 27960 and proceeds from left to right (Supplementary Figure S4D). Packaging of a whole-genome equivalent must be followed by the introduction of a double-stranded break at nucleotide position 27 960 of the previously published genome and the addition of a 6639 bp long LTR. The LTR coincides with the area from which spacers are actively selected.

### The apparent bias in spacer acquisition from the phiFa genome is due to selection of phage-resistant bacterial clones

The observed extreme positional and strand bias of spacers acquired during phiFa infection (Figure 2C) could reflect a true bias of Type III adaptation machinery towards a narrow region of a phage genome. Alternatively, the bias can be an indirect result of selection of bacteria that are resistant to the virus. By analogy with phage T5 (71), it is highly likely that phiFa LTR positioned on the left-hand side of the genome map shown in Figure 2B is injected into an infected cell first. If spacers uptaken from this end at the earliest stages of the infection provide resistance to the phage, the corresponding clones will become more abundant in the course of infection. The second scenario is supported by the analysis of distribution of (i) spacers mapping outside the early region of the phage genome and (ii) unique phage-derived spacers, when abundance of spacers is not taken into account. As can be seen from Figure 2D and Supplementary Figure S5, spacers targeting middle and late genes of the phage mapped to both transcribed and non-transcribed strands, while unique spacers mapped uniformly over the entire phage genome without a strong strand bias. In 3.2% of sequencing reads the amplified region of CRISPR array contained two new spacers acquired during phiFa infection. Spacers from these reads were mapped to the ‘cold’ region of the phage genome more frequently than expected from the distribution of single spacers (*P*-value < 10^–16^, chi-square test). Moreover, 98% of spacers mapped to the ‘cold’ region among reads with two spacers were leader-proximal, i.e. were acquired in CRISPR array after the acquisition of a spacer from a hot region. These observations suggest that an addition of spacer from ‘hot’ region increases the chances of acquisition of the second one from the ‘cold’ region, consistent with an idea that the bias in acquired spacers is selection driven.

**Figure 3.**
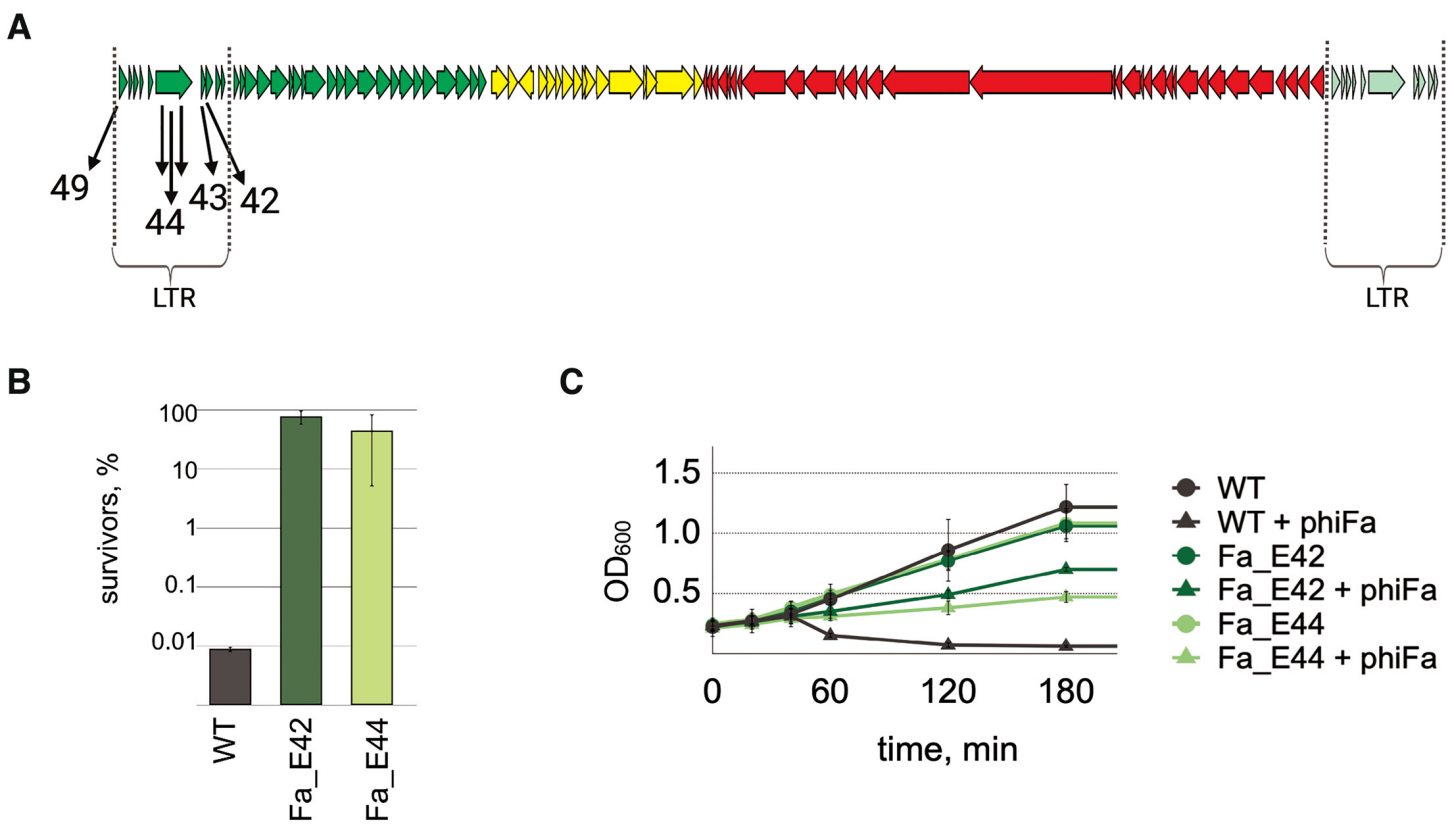
*T. thermophilus* cells carrying Type III spacers targeting early phiFa genes are resistant to infection. (**A**) Spacers acquired by *T. thermophilus* HB27c clones recovered after phiFa infection are shown below a schematic representation of the phage genome. Spacer numbers correspond to the numbers of phage ORFs from which the spacers were acquired (see also Supplementary Table S4). (**B**) Survival of phiFa infection by wild-type *T. thermophilus* HB27c and strains that acquired spacers targeting indicated phage genes. Bars demonstrate percent of colony-forming units in indicated cultures 60 min post-infection. Colony-forming units in control non-infected cultures were taken as 100%. Mean values and standard deviations obtained from three replicas are presented. (**C**) Growth of phiFa infected and uninfected wild-type *T. thermophilus* HB27c and strains carrying indicated spacers. Mean values and standard deviations obtained from 3 replicas are presented.

Several individual *T. thermophilus* clones with CRISPR-2 and/or CRISPR-11 arrays expanded by spacers acquired from the ‘hot’ region of the phage genome were randomly recovered from infected cultures (Figure 3A, Supplementary Table S4) and their ability to withstand the infection was tested by determining EOP (efficiency of plaquing) of the phage. As can be seen from Supplementary Figure S6, acquired spacers indeed provided strong (∼5 orders of magnitude) protection from infection compared to bacteria with unexpanded arrays.

We next determined whether cells carrying protective spacers survived the infection. At conditions of high MOI, when on average every cell was infected by five phage particles at the beginning of the experiment, only 0.01% of cells survived and formed colonies. By contrast, at the same conditions ∼50% of infected cells carrying phage-derived spacers formed colonies (Figure 3B).

In another experiment, the growth of cells infected at MOI of 5 was monitored in liquid cultures (Figure 3C). Cultures without phage-targeting spacers collapsed shortly after the infection. By contrast, infected cells with protective spacers continued to grow, though the optical density of infected cultures was considerably lower than in uninfected controls. We attribute this decrease to the appearance of phage escaper mutants (see below). Overall, we conclude that many cells carrying spacers derived from LTR survive the infection and are cured of the phage. Therefore, the Type III CRISPR–Cas immunity in *Thermus* cultures is not determined solely by the suicidal death of infected cells.

**Figure 4.**
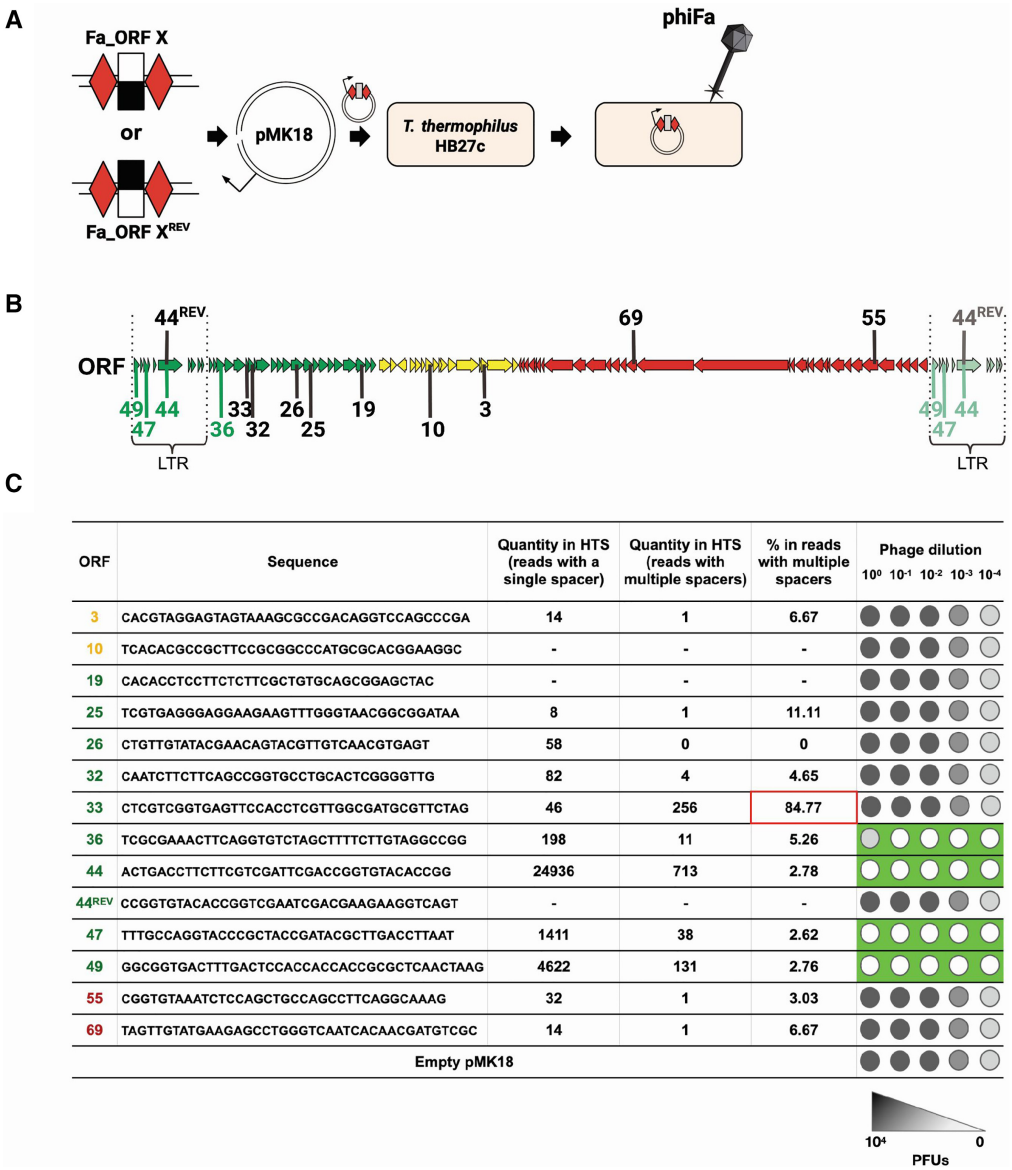
Type III spacers targeting the phiFa genome outside the “hot" region do not protect from infection. (**A**) A dsDNA fragment coding a desirable spacer sequence targeting transcribed or non-transcribed strand of phage genes (white/black rectangle) flanked by Type III repeats (red rhombi) is inserted into the pMK18 plasmid. *T. thermophilus* HB27c cells are transformed with mini-array bearing plasmid and tested for ability to withstand phiFa infection. (**B**) A scheme of the phiFa genome with protospacers matching spacers tested in plasmid-borne mini-arrays is presented. Spacers are numbered according to phage gene they are derived from. Spacers matching protospacers marked with green color protected cells from infection, spacers matching protospacers marked in black – did not. (**C**) Sequences of spacers targeting indicated phiFa genes that were chosen to check for phage protection in a plasmid-based assay. The ORFs from which spacers derived are color-coded to show the expression classes (panel **B**). Quantities of Illumina reads corresponding to each spacer in expanded arrays of cultures after phiFa infection (see Figure 2C) are shown separately for reads containing one or multiple acquired spacers. Spacer 33 targeting an early gene outside the LTR fails to protect the cells when placed on a plasmid-borne mini-array. This spacer was found together with a spacer targeting the LTR-located gene *40*, which while not tested separately, is likely to be protective and was the most abundant of all acquired spacers (found in 138 494 reads). The ability of plasmids bearing mini-arrays with these spacers to protect against phiFa infection is schematically presented in the rightmost column (primary data are shown in Supplementary Figure S7).

Since cells carrying spacers derived from middle and late genes of the phage could not be isolated from infected cultures, we constructed pMK18-based plasmids carrying artificial Type III mini-arrays with a single anti-phiFa spacer flanked by two repeats (Figure 4A). Several plasmids carrying spacers targeting transcripts of various regions of the phage genome were prepared (Figure 4B). A plasmid bearing a mini-array with ‘early’ spacer E44 that targeted the non-transcribed strand (and, therefore, the mRNA) of phage gene *44*, was frequently acquired during the infection and provided phage resistance when carried in CRISPR-2 (Supplementary Figure S6) served as a control. As another control, a plasmid with a mini-array carrying spacer 44^REV^ targeting the opposite, transcribed, strand of gene *44* was used. Cultures of wild-type *T. thermophilus* HB27c cells carrying different mini-array plasmids were tested for their ability to withstand phiFa infection. As can be seen from Figure 4C and Supplementary Figure S7, a plasmid bearing the E44 spacer made cells resistant to infection, indicating that the plasmid-based mini-array is functional. Cells harboring a plasmid targeting the opposite strand of gene *44* were fully sensitive to the phage, demonstrating that Type III interference is transcription-dependent, as expected. Cells targeting transcripts of genes *47* and *49* from the hot region were resistant. Cells bearing a plasmid with a spacer targeting a transcript of gene *36*, which is located outside the LTR at the border of the ‘hot’ and ‘cold’ phage genome regions, were partially resistant (an EOP ca. order of magnitude less than for cells carrying spacers targeting transcripts of genes *44*, *47* and *49* located within the LTR). All cells carrying mini-arrays with spacers targeting early genes further outside of the hot region or middle/late genes were fully sensitive to the phage.

Using the plasmid-based interference assay we ascertained that acquired spacers indeed protect cells from phiFa infection through the action of Type III CRISPR–Cas systems of *T. thermophilus* HB27c. To this end, a plasmid bearing the protective E44 spacer was transformed into previously described derivative strains lacking Type III-A, Type III-B or both effector complexes (35) and susceptibility to phiFa was determined. As can be seen from Supplementary Figure S8, cells lacking both effectors were fully sensitive to the phage in the presence of E44 spacer carrying plasmids, while the wild-type and both single mutants were resistant. We therefore conclude that acquired spacers can be used by either III-A or III-B subtypes to protect the cells from phage infection.

To test whether crRNA with spacers targeting middle/late genes are functional in CRISPR interference, plasmids simultaneously carrying a mini-array and a matching transcribed protospacer were created. We expected that such ‘self-targeting’ plasmids will not be efficiently transformed in the host. Indeed, the presence of a plasmid-borne protospacer matching the mini-array spacer decreased transformation efficiency (Supplementary Figure S9). This effect was observed for plasmid-borne spacer-protospacer pairs from early, middle, and late genes of the phage. We, therefore, conclude that the observed positional and strand bias in acquisition of spacers during phiFa infection is caused by counter-selection against cells that acquired non-functional spacers from outside the early region rather than by an intrinsic bias of the Type III adaptation machinery.

### The phiFa phage can escape the action of Type III CRISPR–Cas system

**Figure 5.**
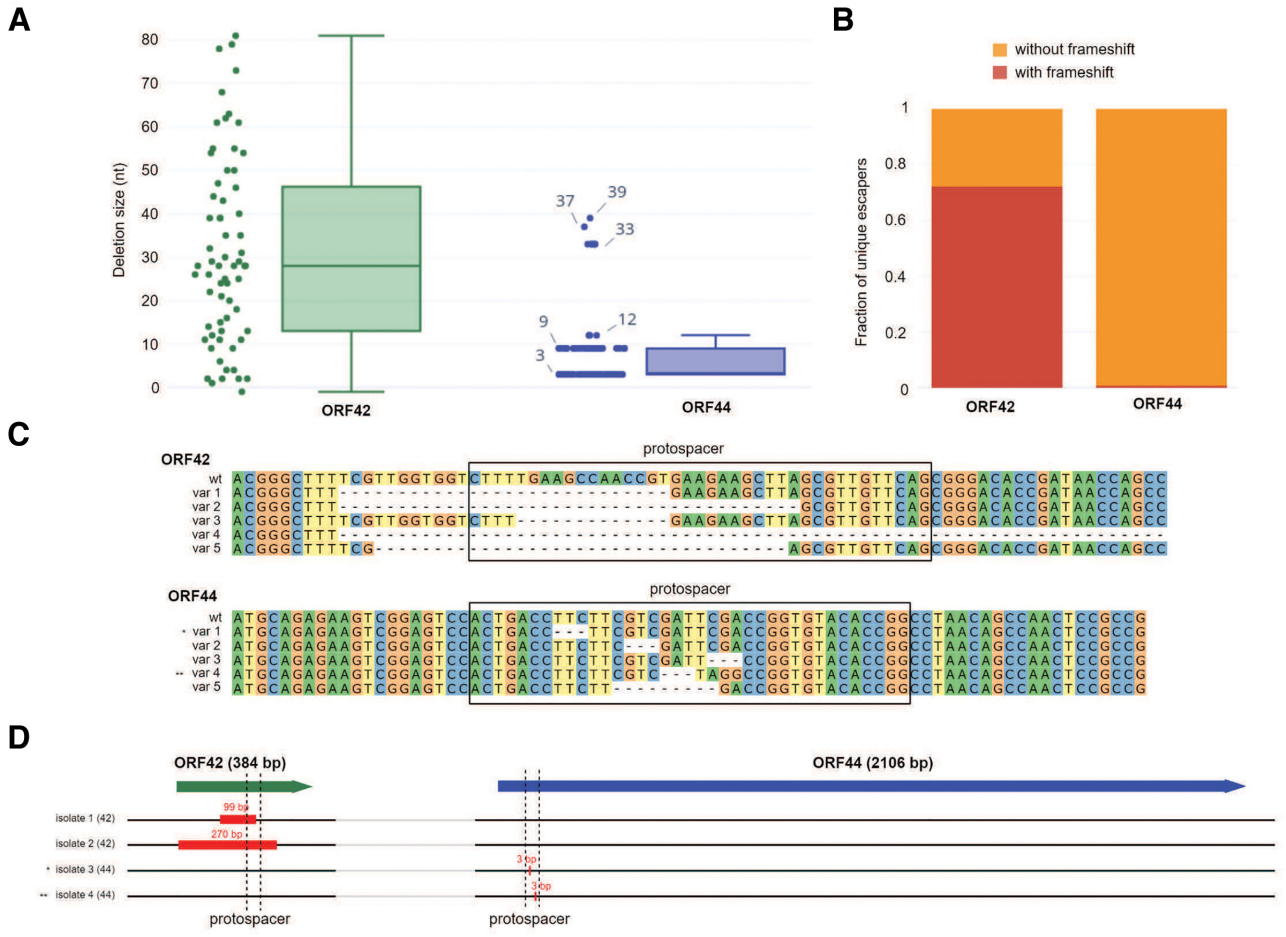
The phiFa bacteriophage escapes Type III CRISPR–Cas action by accumulating deletions in targeted protospacers. (**A**) Comparison of deletion sizes in targeted regions of phiFa genes *42* and *44* in cell-free supernatants of infected cultures protected with corresponding spacers. For each gene, analysis of HTS data are shown as box-plots and as scatter plots, with each point representing a unique phiFa sequence encountered in supernatants of infected cultures of cells harboring spacers targeting the corresponding gene. (**B**) Frequency of frame-shifting mutations in unique phiFa sequences encountered in supernatants of infected cultures of cells harboring spacers targeting genes *42* and *44*. (**C**) Five most abundant deletions in phiFa escaper phages detected by HTS sequencing of protospacer regions located within genes *42* and *44*. Positions of protospacers are highlighted with black boxes. (**D**) Deletions detected in four phiFa escaper phages (isolates ‘1’ and ‘2’ were obtained from gene *42* targeting culture, isolates ‘3’ and ‘4’ – from gene *44* targeting culture). Genes *42* and *44* are shown at the top as green and blue block arrows, whose lengths are proportional to the lengths of the genes. Location of protospacers within the genes is shown with dashed lines, the size of detected deletions - with red rectangles.

Some cultures of cells with protective spacers collapsed after overnight growth in the presence of the phage suggesting that escaper phages may accumulate in the course of infection. Indeed, cell-free supernatants of collapsed cultures lysed cells resistant to the wild-type phage (Supplementary Figure S10). To determine the spectrum of mutations present in escaper phages, regions of phage DNA around two targeted protospacers, in gene *44* coding for phage RNA polymerase (RNAP) and in gene *42* encoding a hypothetical protein, were amplified from the supernatants of corresponding collapsed cultures and subjected to HTS. While ∼50% of reads contained wild-type targeted protospacers, the rest harbored deletions. In gene *44*, deletions had lengths equaling multiples of three nucleotides preserving the reading frame of the presumably essential viral RNAP gene (Figure 5A and B). In gene *42*, only one third of recovered deletions preserved the reading frame, suggesting that gene *42* product is not essential for the phage (Figure 5A and B). Deletions in this gene were also longer than in gene *44* (Figure 5С). To verify that detected mutations are indeed present in infectious viral particles, several escaper phages were isolated on lawns of cells carrying gene *44*- or gene *42*-targeting spacers and their full genome sequences were determined. In addition to 0–3 random point mutations detected in each sequenced isolate (Supplementary Table S5), deletions in corresponding CRISPR-targeted regions were found. In agreement with the HTS analysis of amplicons, two escapers selected on gene *44*-targeting lawn harbored 3-bp deletions, while 99- and 270-bp deletions were observed in phages that escaped gene *42-*targeting cells (Figure 5D). We conclude that phages can escape Type III CRISPR–Cas defense by small deletions that maintain the reading frame (and presumably, the function) of essential genes or by much larger deletions in nonessential genes.

### Type III spacers acquired from all temporal classes of phage phiKo genes protect cells from infection

**Figure 6.**
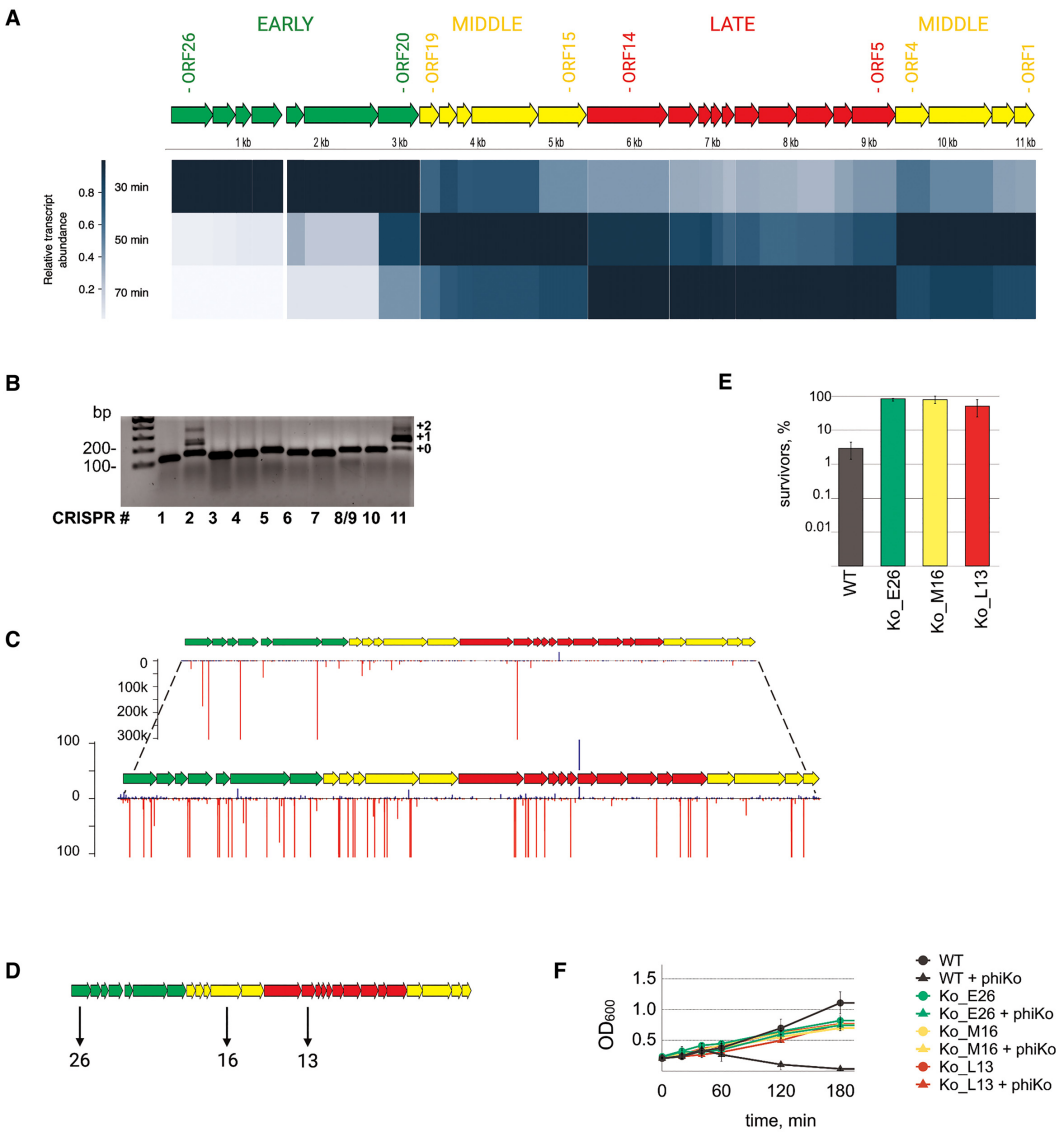
*T. thermophilus* cells carrying Type III spacers targeting each class of phiKo genes are resistant to infection. (**A**) Analysis of transcription of phiKo genes during the infection processes. The scheme of the phiKo genome is depicted. Predicted ORFs are marked as arrows and colored according to their temporal classes. Early, middle and late genes are colored in green, yellow and red, respectively. The heat map indicates the transcript abundance for each gene normalized to the maximum transcript abundance for this particular gene. (**B**) Expansion of *T. thermophilus* HB27c CRISPR arrays in a culture infected with phiKo phage. Lane numbering and the method of CRISPR expansion assay are the same as described for Figure 1B. (**C**) Mapping of spacers acquired by CRISPR-2 and CRISPR-11 arrays on the phiKo genome. Spacers whose sequences match the ‘top’ strand of phage DNA (5′-3′ direction) are shown as blue bars, those matching the ‘bottom’ strand – as red bars. The height of a bar reflects the quantity of reads corresponding to a particular spacer. The lower views for each array allow one to see minor spacers (scales indicating the number of reads are indicated on the left). All spacers from CRISPR-2 and CRISPR-11 arrays were combined before mapping. (**D**) Spacers acquired by three of *T. thermophilus* HB27c clones recovered after phiKo infection are shown below a schematic representation of the phage genome. (**E**) Survival of phiKo infection by wild-type *T. thermophilus* HB27c and strains that acquired spacers targeting indicated phage genes. Bars demonstrate percent of colony-forming units in indicated cultures 60 min post-infection. Colony-forming units in control non-infected cultures were taken as 100%. Mean values and standard deviations obtained from 3 replicas are presented. (**F**) Growth of phiKo infected and uninfected wild-type *T. thermophilus* HB27c and strains carrying indicated spacers. Mean values and standard deviations obtained from three replicas are presented.

Earlier, a *Tectiviridae* family phage phiKo infecting *T. thermophilus* HB27c was isolated in our laboratory (44). Since phiKo is unrelated to phiFa, it was of interest to determine whether results obtained with phiFa with regards to Type III CRISPR immunity apply to phiKo. To this end we first performed RNA-seq of phiKo-infected cultures to reveal temporal classes of phage genes. Aliquots of phiKo infected *T. thermophilus* HB27c cultures were collected before infection (as a control) and 10, 30, 50 and 70 min post-infection (Supplementary Figure S11A). RNA-Seq reads were mapped on the host and phage genomes (Supplementary Figure S11B) and three temporal classes of phage genes were distinguished by following transcript abundance of each phage gene throughout the infection (Supplementary Figure S11C, Figure 6A). ORFs *20–26* represent the ‘early’ class; ORFs *1–4* and ORFs *15–19* represent the ‘middle’ class; ORFs *5–14* represent the ‘late’ class of phiKo genes (Figure 6A).

We next monitored CRISPR arrays expansion in phiKo-infected cultures using the protocol developed for phiFa infection. As can be seen from Figure 6B, the result obtained during phiKo infection was the same as that seen with phiFa: only Type III CRISPR-2 and CRISPR-11 were expanded. Amplicons corresponding to the expanded arrays were purified and subjected to high-throughput sequencing and the data was analyzed as described above. The analysis revealed that more than 98% of acquired spacers were obtained from the phage and most of phiKo-originating spacers targeted the non-transcribed strand of phage genes (Figure 6C). If only unique spacers were considered, the strand bias vanished (Supplementary Figure S12), as was also the case for phiFa-originating spacers (above). However, in contrast to phiFa, the distribution of spacers across the phiKo genome was more even and spacers originating from genes of every temporal class were detected in significant numbers (Figure 6C).

Several individual *T. thermophilus* clones with expanded CRISPR-2 or CRISPR-11 arrays were isolated from infected cultures (Supplementary Table S4) and clones that acquired spacers from early, middle, and late phiKo genes were selected among them and shown to provide resistance on plates in drop assays (Supplementary Figure S13). The resistance was due to the action of Type III CRISPR–Cas systems of *T. thermophilus* HB27c, since the Δ*cmr4* Δ*csm3* double mutant carrying a plasmid with a mini-array with protective spacer was fully sensitive to phiKo, while the wild-type strain and single mutants were resistant (Supplementary Figure S14). We next determined whether cells carrying protective spacers against phiKo survived the infection (Figure 6D). As can be seen from Figure 6E, up to 100% of cells from carrying phage-derived spacers formed colonies after high-MOI infection (Figure 6E) and the growth rate of cultures of non-infected and infected cells was very similar (Figure 6F). Thus, cells mounting Type III interference against the phiKo phage survive the infection.

## DISCUSSION

Components of the III-A and III-B CRISPR–Cas system subtypes in *T. thermophilus* were extensively studied *in vitro* by biochemical and structural methods (22,75,76). However, investigations of the biological function of these systems are limited. In this work, we have demonstrated the ability of *T. thermophilus* Type III CRISPR–Cas system to provide robust resistance to phage infection through accumulation of spacers targeting phage transcripts arising from a narrow region of the lytic phiFa phage genome. Previously, Type III adaptation was only detected when adaptation machinery components were overexpressed (39,40). Moreover, in published work Type III CRISPR–Cas systems encoding RT-Cas1 fusion proteins were studied. Though such fusions allow acquisition of spacers directly from RNA, only ∼8% of bacterial Type III CRISPR–Cas systems bear them; the rest encode ‘standard’ Cas1 proteins (39).

In the present study, we explored adaptation by a Type III system with conventional Cas1 lacking the RT domain at ‘natural’ conditions, i.e., without overexpression of CRISPR–Cas systems components. The use of virulent phages provided a strong selection pressure to enrich cells that acquired protective spacers. Most of the acquired spacers detected in *T. thermophilus* population after the infection with phiFa originated from a narrow segment of the early phage genome region and were complementary to phage transcripts - a required condition for RNA-dependent Type III interference. Spacers acquired during the infection by an unrelated phiKo phage were more randomly distributed along the phage genome, but also showed strong strand bias, targeting phage transcripts. In cultures infected by both phages, we also detected a small fraction of spacers mapping to *T. thermophilus* chromosome and megaplasmid. Mapping of these spacers did not reveal strong biases in strand distribution. This observation suggests that the observed very strong strand bias in distribution of phage-derived spacers is a consequence of selection for functional spacers that protect cells from the phage, rather than a result of activity of a special mechanism that channels acquisition of spacers from transcripts, like in systems encoding RT-Cas1 proteins.

Individual bacterial clones with Type III arrays expanded by spacers targeting the non-transcribed strand of early phiFa genes were resistant to the phage. In contrast, expression of crRNA targeting the transcribed strand did not provide phage resistance. It thus follows that 50% of spacers acquired by *T. thermophilus* Type III and, by extension, by other Type III systems relying on Cas1 lacking an RT domain, may not be able to provide defensive function to the cell. We also found no correlation between spacers acquired in biological replicas or individual Type III CRISPR arrays. The result suggests that the Type III adaptation machinery is selecting spacers randomly, a mechanism that is made possible by the absence of PAM requirement for target interference by these systems. As a result, the maximal ∼50% efficiency of acquired Type III spacers is comparable to reported numbers of ∼40–50% of interference-proficient spacers acquired during naïve adaptation by PAM requiring Type I systems (8,77).

The vast majority of incorporated spacers detected in infected cultures target a set of early phiFa genes located in the long terminal repeats. These spacers also protect the cells from the phage. Spacers targeting phage RNA transcribed from outside the LTRs (or immediately adjacent region of the phage genome) do not provide resistance. While we did not expressly show this, we assume that protective spacers target the copy of LTR that is inserted first into infected cells. A similar situation was observed by us earlier while studying Type I-E mediated defense from *E. coli* phage T5, whose genome also contains LTRs. In the case of T5, the LTR carries several genes whose products are cytotoxic and are responsible for host takeover, destroying cell DNA and making it serve as a mere factory for production of phage progeny (75). As a result, cells targeting T5 LTR do not survive the infection but the population as a whole benefits from the altruistic death of infected cells that mount a CRISPR interference response. In the case of phiFa infection, the situation is different, since most infected cells targeting LTR transcripts survive. It, therefore, follows that phiFa, or more precisely, its LTR, either does not encode cytotoxic products or the synthesis of these proteins is effectively prevented by Type III immunity. Why are then spacers targeting transcripts from the rest of the phage genome, which undoubtedly contains essential genes, not detected in cells surviving the infection? One possibility would be that one of the LTR genes encodes an anti-CRISPR protein(s) that specifically inhibits Type III adaptation, Type III interference, or both. A precedent for this is known: a non-specific auxiliary RNase Csm6 is required for Type III immunity during targeting of late viral transcripts in *Staphylococcus epidermidis* (34). A type III CRISPR–Cas system phage-encoded inhibitor, which suppressed the activity of the Csx1 RNase abrogated the immune response directed towards middle/late but not early phage transcripts (78). The phiFa LTR encodes 8 small (less than 150 amino acids) proteins with unknown function and it would be interesting to test their activity on Type III immunity. Another, perhaps more radical, idea explaining the bias in protective spacer distribution is related to the transcription strategy of phiFa and its relatives. These phages encode a single-subunit RNA polymerase of their own, whose gene is located in the LTR (44). Simple, single-subunit RNAPs are known to elongate faster than multisubunit host enzymes (79). If phiFa RNAP also possesses this property, it may be able to escape from co-transcriptional Type III targeting tuned towards nascent transcripts produced by the slower host transcription apparatus. The influence of phiFa physiology on the pattern of spacer acquisition is supported by the fact that in the case of infection by an unrelated phage phiKo, spacers targeting various regions of the phage genome are distributed much more uniformly.

Given that *invitro* interaction of Type III effectors with their targets CRISPR–Cas activates non-specific degradation of both target and non-target RNA and DNA, *in* *vivo* Type III immunity may cause cell growth retardation, dormancy or even death, acting as abortive infection (ABI) rather than specific immunity system. The nature of Type III CRISPR–Cas systems also allows the involvement of additional cyclic oligoadenylate-responsive mechanisms that may cause cell death/growth retardation. Indeed, it was shown that the non-specific RNase activity of the Csm6 nuclease during type III-A mediated immune response induces growth arrest of *S. epidermidis* cells; but this activity by itself does not provide immunity against plasmid conjugation (80). The nuclease domain of Cas10 activated upon the binding to target RNA by the Csm/Cmr complexes possesses a ssDNase activity, which, however, does not appear to have a noticeable negative effect on cell growth (80). Recently, a non-specific DNase NucC, which is activated by cyclic oligoadenylate, was discovered (81). NucC is a component of an abortive infection system that causes cellular DNA degradation and cell death upon infection. It was also shown that NucC homologs are associated with type III CRISPR–Cas systems (82). Intriguingly, a Type III CRISPR–Cas system in *Serratia* encoding a NucC homolog was shown to provide immunity against PCH45 phage that physically blocks access of CRISPR–Cas effectors to its DNA during infection. The NucC nuclease is necessary for protection against PCH45, suggesting that it may degrade cellular DNA and cause cell death upon infection (82). All this evidence notwithstanding, our work clearly shows that Type III CRISPR–Cas systems in *T. thermophilus* endow cells with *bona fide* immunity, i.e. cells mounting a Type III immune response survive the infection. The number or survivors ranges from ∼50% in the case of phiFa-infected cells to almost 100% in the case of phiKo infection. The result indicates that the developmental strategy of the phage may significantly influence the number of surviving cells mounting the Type III immunity, which needs to be taken in account when interpreting the sometimes conflicting *in vivo* data on Type III interference.

Survival of *T. thermophilus* cells mounting the Type III response may be accounted by the activity of ring nucleases capable of degrading cyclic oligonucleotides (83). The activity of these nucleases is thought to switch-off non-specific RNases allowing cells to recover from growth arrest and avoid death. Since *T. thermophilus* with protective Type III spacers clear the virus, the double-stranded phage DNA must be degraded before the cell succumbs to infection. Which component of Type III system is responsible for phage DNA degradation remains to be determined. Three CARF-domain proteins are encoded in the *T. thermophilus* HB27c genome by HB27c_P0152 (CARF^1^), HB27c_P0154 (CARF^2^) and HB27c_P0142 (CARF^3^) genes. Very close homologs (99% identity) for all three genes can be found in *T. thermophilus* HB8 (TTHB144, TTHB155 and TTHB152) and were functionally characterized *in vitro* (84,85,32). We used available information about these homologs and classification of CARF proteins (31) to predict the function of CARF proteins in *T. thermophilus* HB27c. The following results were obtained. The CARF^1^ protein contains a cA4-activated HEPN ribonuclease domain. The CARF^2^ protein contains a DNase domain (restriction endonuclease fold), which has specificity to supercoiled ssDNA. The CARF^2^ activity can decrease the replication rate of phage DNA (ssDNA nicks will cause the collapse of replication forks) while leaving slowly-replicating host chromosome relatively unaffected. The CARF^3^ protein contains an active HEPN ribonuclease domain, and a specialized CARF domain, which has been shown to degrade cA4. Proteins with the same domain composition (pfam09670_CARF_6H_HEPN) are associated with type III-A and type III-B systems in Bacteria and Archaea but have not been found in *Staphylococcus* studied by the Marraffini group (34,80,86). It is thus possible that that infected *Thermus thermophilus* cells mounting Type III interference can recover from growth arrest caused by collateral RNA destruction through CARF^3^-mediated degradation of cA4.

While *T. thermophilus* HB27c harbors seven Type III CRISPR arrays with identical repeat sequences, active spacer acquisition was demonstrated only for CRISPR-2 and CRISPR-11 arrays. According to RNA-seq data (Supplementary Table S6), all Type III arrays, with the exception of CRISPR-1, which lost the leader sequence through transposon integration, are transcribed at comparable levels and so the apparent lack of adaptation is unlikely to be due to the inability of spacers acquired in these arrays to provide protection from phage infection. Phylogenetic analysis of leader sequences revealed two clades comprising CRISPR-2, CRISPR-11 and CRISPR-5 in one clade and the three remaining arrays in the other clade (Supplementary Figure S15). Sequence differences common to leaders of the latter clade may be responsible for the lack of spacer acquisition, though this can not explain the lack of acquisition in CRISPR-5. The two acquisition proficient arrays are the only ones located in close proximity of Type III interference (CRISPR-2) or the *cas1*^4^ (CRISPR-11) gene required for adaptation (Figure 1A). Interestingly, in addition to truncated *cas1*^2^ and *cas1*^3^ sequences located upstream CRISPR-8 and CRISPR-10 (Figure 1A), sequences corresponding to even shorter regions of 3′ region of *cas1* genes are present upstream of CRISPR-5 and CRISPR-9. Thus, rudimental pieces of *cas1* genes exist upstream of leaders of each of the four Type III CRISPR arrays incapable of spacer acquisition in our system (Supplementary Figure S16). Whether these gene fragments encode proteins or can affect spacer acquisition *in cis* by some unknown mechanism remains to be determined. Variations in leader sequences and the presence of partial *cas1* genes suggest that *cas1*^4^, CRISPR-2 and CRISPR-11 co-evolved independently from the rest of Type III CRISPR arrays (87).

Although *T. thermophilus* HB27c encodes not only Type III Cas1 but also a I-B subtype homolog, robust adaptation in the presence of phiFa phage was detected only for Type III arrays. In our previous work on *Thermus* environmental communities (44), we demonstrated that spacers originating from phiFa and related viruses were only found in Type III but not Type I CRISPR arrays. The Type I CRISPR arrays of *T. thermophilus* HB27c are transcribed (Supplementary Table S6). These observations may indicate phiFa and its relatives have a special mechanism to resist the action of Type I CRISPR–Cas systems. It is worth noticing that most of environmental spacers targeting phiFa were also mapping to the transcribed strand of phage LTR, indicating that they are protective and that the adaptation process detected in the laboratory mimics that happening in natural *Thermus* communities.

Previously, it was reported that the high tolerance of Type III system to mutations in protospacer and the absence of PAM limit accumulation of viral escapers (86). Escaper phages in type III CRISPR–Cas systems were obtained in case of targeting of transcripts of a nonessential gene. It was shown that such escapers harbor deletions spanning the protospacer. However, attempts to obtain escapers while targeting transcripts of essential genes were unsuccessful. Recently, phages which escaped Type VI CRISPR–Cas immunity were detected. The isolates had extended (up to ∼3 kb) deletions surrounding protospacer areas that preserved the reading frames of targeted genes (81,88). Our with phiFa escapes corroborate these observations. The simple plasmid-based crRNA production system described here allows one to easily obtain both large and small deletions in *Thermus* genome and in the genomes of phages susceptible to Type III immunity, opening ways for functional analysis of host and viral genes without applying the homologous recombination step.

## DATA AVAILABILITY

Raw sequencing data have been deposited with the National Center for Biotechnology Information Sequence Read Archive under BioProject ID PRJNA631468.

## Supplementary Material

gkaa685_Supplemental_File
