# Supplementary material for: Spacer acquisition by Type III CRISPR–Cas system during bacteriophage infection of Thermus thermophilus
Source: Nucleic Acids Res. 2020 Aug 21;48(17):9787–803. doi: 10.1093/nar/gkaa685 (PMC7515739; doi:10.1093/nar/gkaa685)
Supplement: gkaa685_Supplemental_File [file gkaa685_supplemental_file.docx]

Supplementary data for

Spacer Acquisition by Type III CRISPR-Cas System During Bacteriophage Infection of *Thermus thermophilus*

Daria Artamonova, Karyna Karneyeva, Sofia Medvedeva, Evgeny Klimuk, Matvey Kolesnik, Anna Yasinskaya, Alexei Samolygo, and Konstantin Severinov

**Supplementary figures**

**
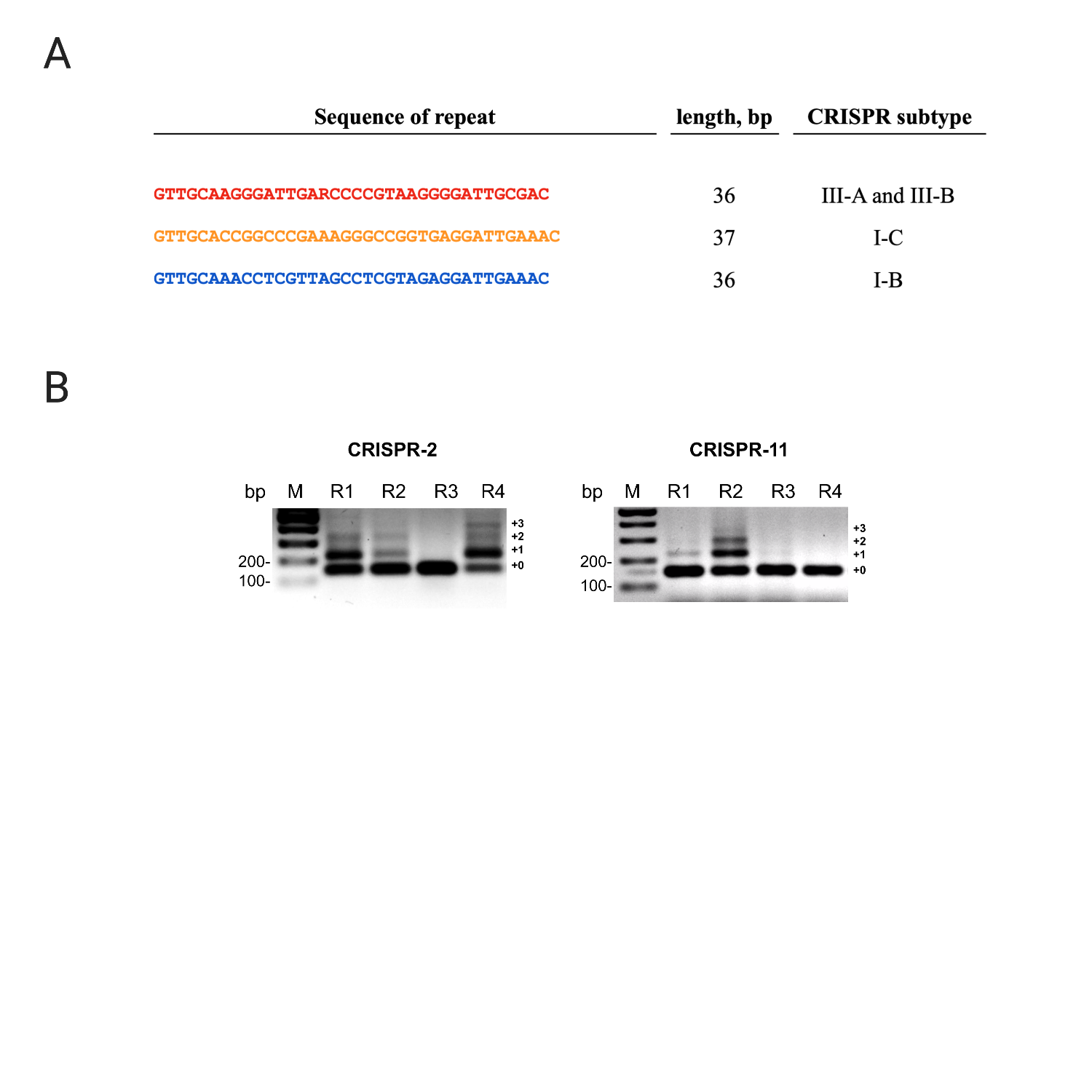
**

**Supplementary Figure S1. Variations in levels of spacer acquisition by CRISPR-2 and CRISPR-11 arrays.**

Spacer acquisition detected in four parallel cultures originating from the same overnight *T. thermophilus* HB27c culture. Individual cultures were infected with phiFa at same OD_600_ and MOI. “M” - DNA ladder, “R1” - “R4” - different replicas. Adaptation was detected the same way as in Figure 1B.


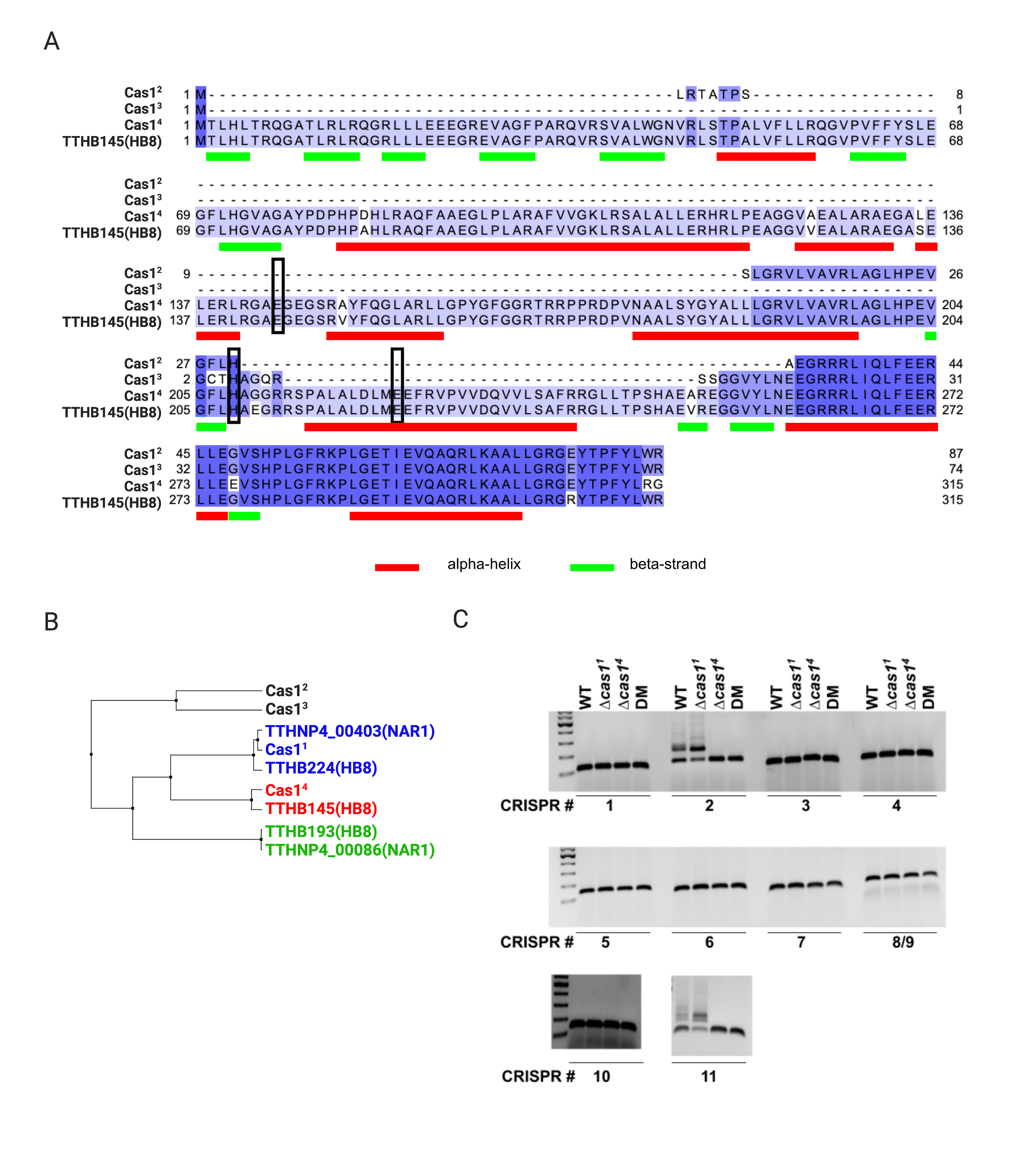


**Supplementary Figure S2. Analysis of sequences and functional activity of Cas1 proteins from *T. thermophilus* HB27c strain.**

**(A)** Amino acid alignment of Cas1 proteins from *T. thermophilus* HB27c and the likely Type III homologue from the HB8 strain. Conserved glutamate and histidine residues coordinating catalytic Mg^2+^/Mn^2+^ions are highlighted in black frames. Stretches of amino acid residues forming alpha-helices (underlined with red) and beta-strands (underlined with green) were predicted with [PROMALS3D](http://prodata.swmed.edu/promals3d/promals3d.php). **(B)** Average distance phylogenetic tree of Cas1 proteins from *T. thermophilus* HB27c (Cas1-1, Cas1-2, Cas1-3, Cas1-4), HB8 (TTHB145(HB8), TTHB193(HB8), TTHB224(HB8)), and NAR1 (TTHNP4_00086, TTHNP4_00403) strains. Colors correspond to CRISPR-Cas system Types: red - Type III, blue - Type I-B, green - Type I-E (average distances were calculated using BLOSUM62 (Jalview 2.10.5)). **(C)** Determination of Cas1 protein essential for Type III spacer acquisition. Expansion of CRISPR arrays in *T. thermophilus* HB27c WT, ∆*cas1^1^*, ∆*cas1^4^*, and ∆*cas1^1^*∆*cas1^4^* double mutant (“DM”) strains during phiFa infection was detected by PCR with primers annealing to leaders and leader-proximal spacers as in Figure 1B.


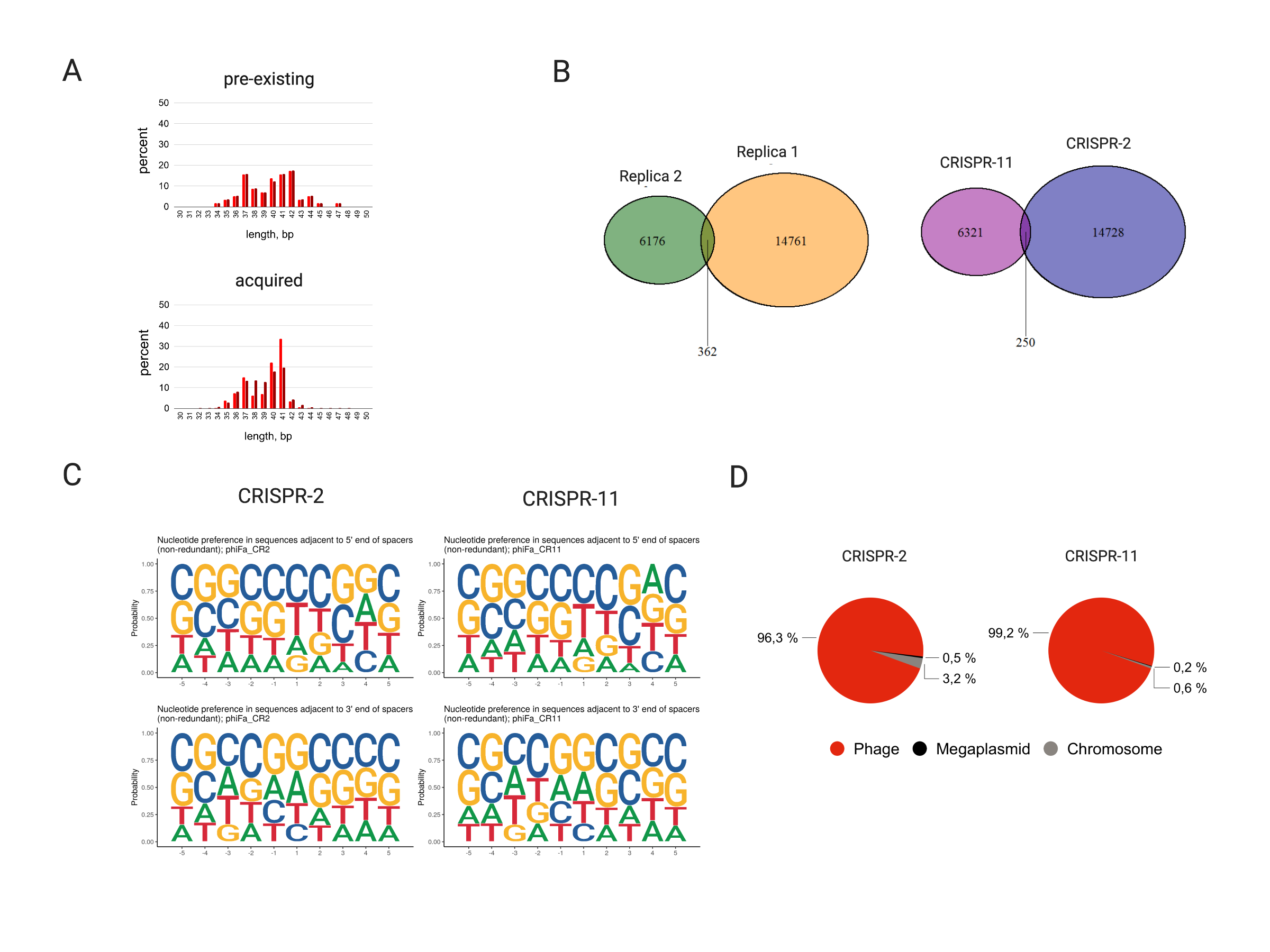


**Supplementary Figure S3. Properties of spacers acquired by CRISPR-2 and CRISPR-11 arrays.**

**(A)** Distribution of spacer lengths in Type III CRISPR arrays. Values were calculated for pre-existing *T. thermophilus* HB27c spacers (top) and newly acquired spacers during infection with phiFa (bottom). Red bars correspond to all spacers, maroon bars – to unique spacers. (**B)** Venn diagrams showing unique spacers acquired by *T. thermophilus* HB27c Type III CRISPR arrays during infection with the phiFa phage. Diversity of spacers in two biological replicates and diversity of spacers acquired by CRISPR-2 and CRISPR-11 are shown. **(C)** Weblogo plots demonstrate the absence of nucleotide preferences in protospacers or protospacer adjacent sequences. Nucleotide sequences in interval +/-5 nucleotides around the 5’ (top panels) and 3’ (bottom panels) ends of aligned spacers were extracted; alignments with identical coordinates were converged to a single alignment. In cases of 5’ end adjacent sequences nucleotide with coordinate 1 represents the 1st nucleotide of aligned spacers; in cases of 3’ end adjacent sequences nucleotide with coordinate -1 represents the last nucleotide of aligned spacers. The height of each letter represents the frequency of the letter at a given position in the set of sequences. Left panel demonstrates results for CRISPR-2 array, right panel - for CRISPR-11. (**D)** The origin of acquired spacers. Spacer sequences obtained after high-throughput sequencing of expanded arrays amplicons were mapped on the phiFa genome and *T. thermophilus* HB27c megaplasmid and chromosome sequences. Fractions of spacers originating from the phage genome are shown in red, from the megaplasmid - in black, and from the chromosome - in grey.


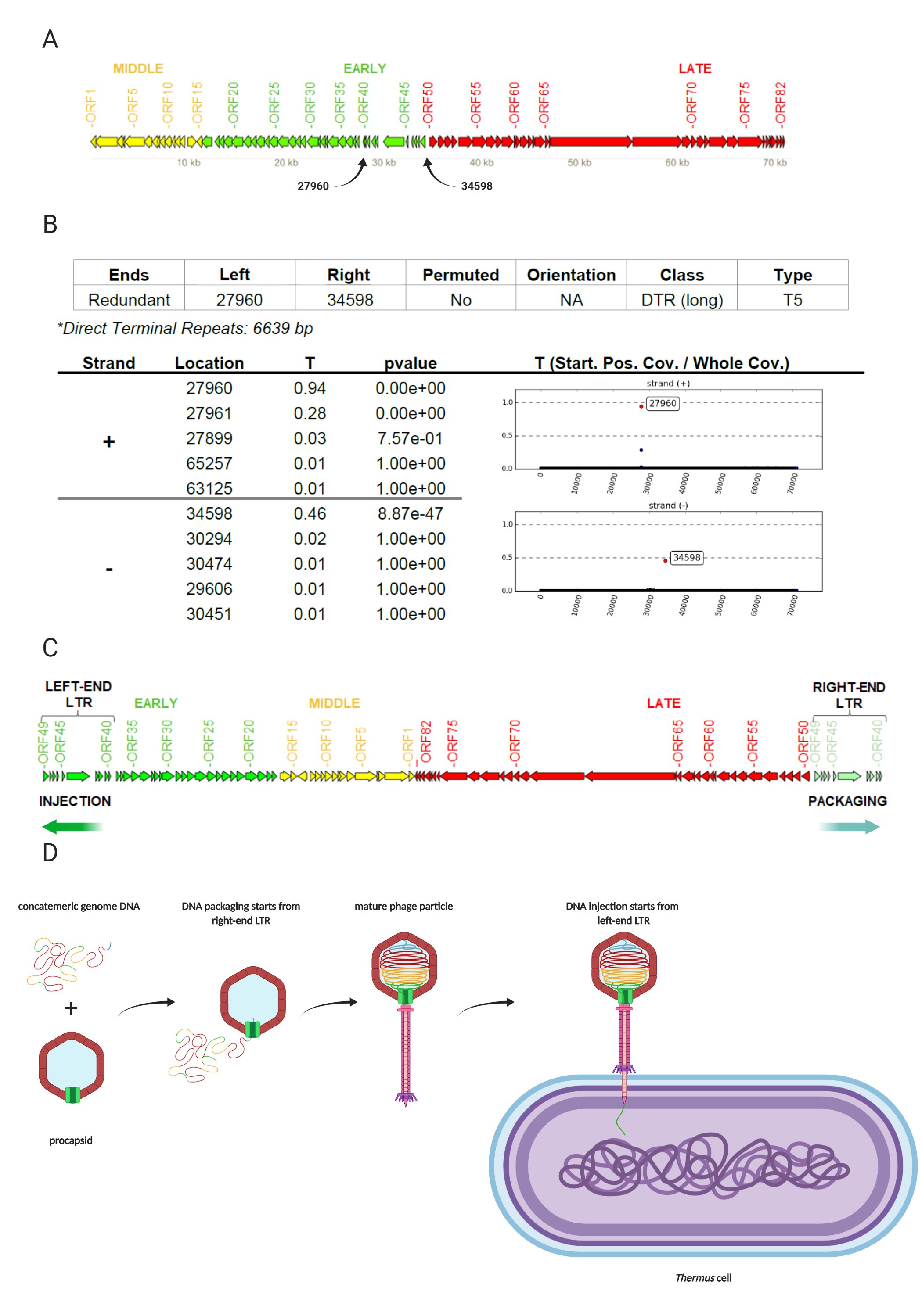


**Supplementary Figure S4. Analysis of phiFa genome termini and a model of the genome packaging process**.

**(A)** Schematic representation of published phiFa genome. Genes are grouped into early (green), middle (yellow) and late (red) classes. The two positions that were determined to be the ends of each genome equivalent are marked. **(B)** Part of PhageTerm tool output file showing main properties of genome termini. Reads are mapped on the reference to determine the starting position coverage (Start. Pos. Cov.) as well overall coverage (Whole Cov.) in each orientation. These values are used to compute the variable T = Start. Pos. Cov. / Whole Cov. The average value of T at positions along the genome that are not termini is expected to be 1/F, where F is the average fragment size. The average value of T at positions along the genome that are termini is expected to be close to 1. **(C)** An updated scheme of the phiFa genome consistent with the PhageTerm tool results. Gene belonging to different expression classes are labeled as in panel **А**. Gene numbering used in the published genome is preserved to avoid confusion. Large terminal repeats (LTR) are marked on both ends of the genome equivalent. The likely directions of genome DNA packaging into the capsid (pale blue/green arrow) and injection to the host cell from the capsid (green arrow) are shown. **(D)** A scheme of concatemer phiFa genome DNA packaging and processing; phage maturation; and injection of phage DNA during infection. Colours of genes correspond to those used in panels **A** and **C**.


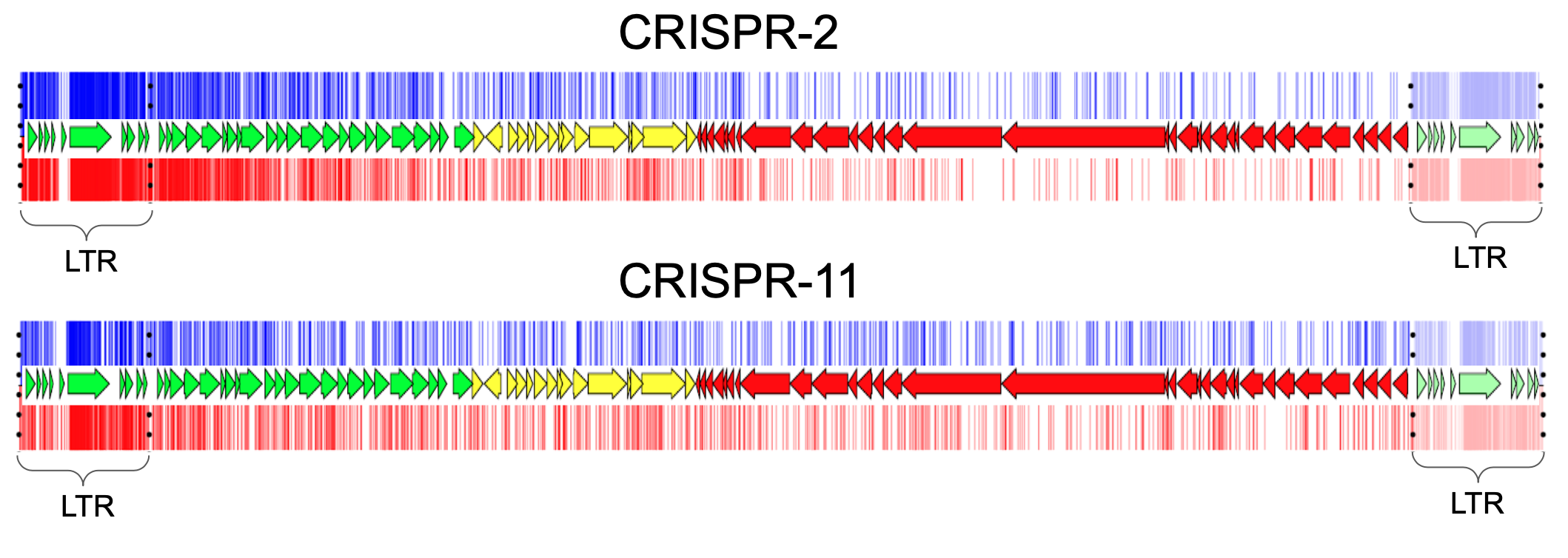
**Supplementary Figure S5. Mapping of unique spacers acquired by CRISPR-2 and CRISPR-11 arrays on the phiFa genome.** Spacers whose sequences match the “top” strand of phage DNA (5’-3’ direction) are shown as blue bars (pale blue - for the right LTR), those matching the “bottom” strand - as red bars (pink - for the right LTR). The frequency with which individual spacers were acquired is not considered in this analysis.


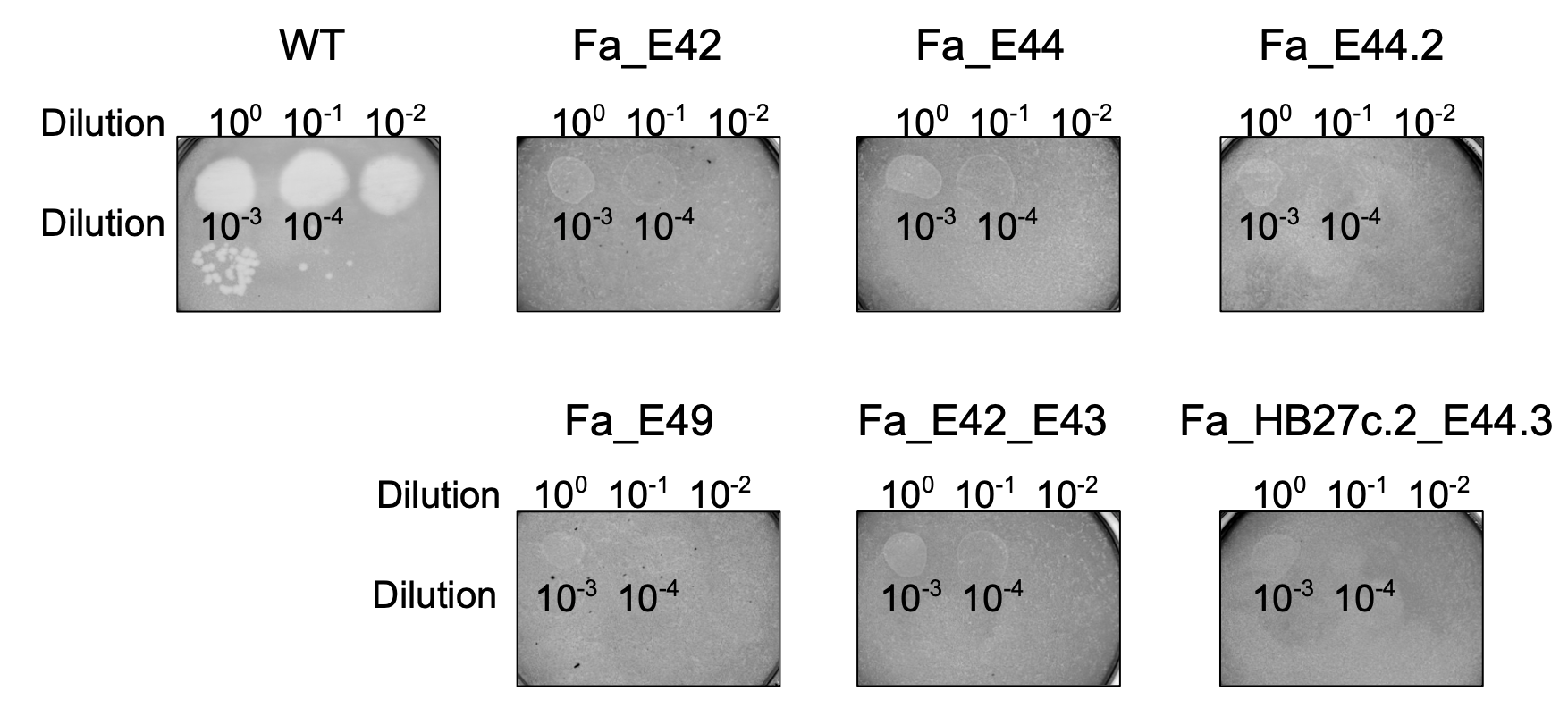


**Supplementary Figure S6. Isolated *T. thermophilus* HB27c strains carrying Type III spacers acquired from phiFa LTR region are resistant to re-infection.**

10-μl aliquots of serial 10x dilutions of phiFa lysate were dropped on lawns formed by wild-type *T. thermophilus* HB27c or lawns of cells isolated from infected cultures that acquired spacers from the indicated early (E) phage genes.

**
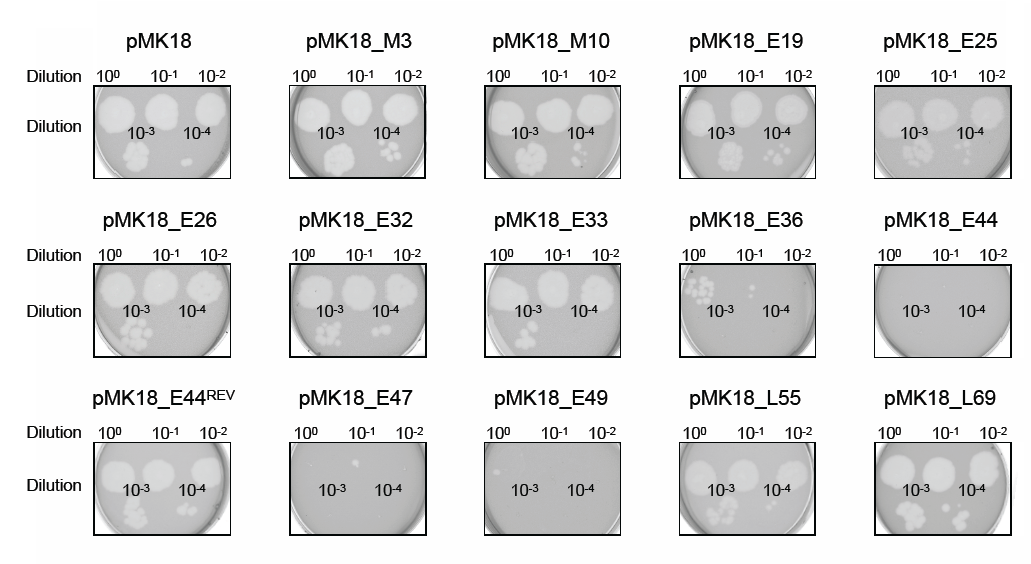
**

**Supplementary Figure S7. The ability of plasmids bearing mini-arrays with indicated spacers to protect against phiFa infection.**

10-μl aliquots of serial 10x dilutions of phiFa lysate were dropped on lawns formed by wild-type *T. Thermophilus* HB27c transformed with the pMK18 plasmids bearing mini-arrays with indicated spacers. Results are schematically summarized in Figure 4C.


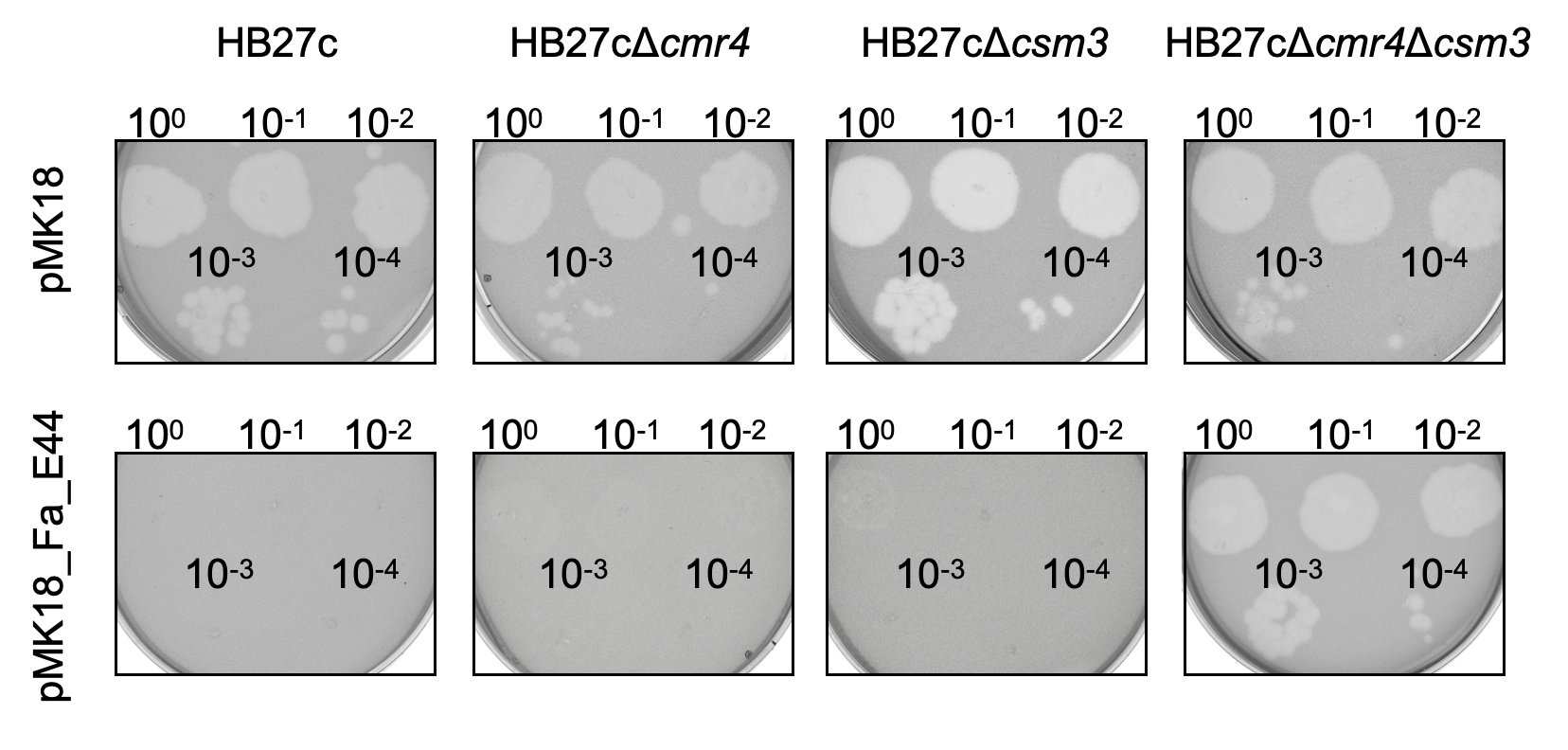


**Supplementary Figure S8. Ability of *T. thermophilus* HB27c WT strain and strains with deactivated III-B (Δ*cmr4*), III-A (Δ*csm3*) or both (Δ*cmr4*Δ*csm3*) interference modules to resist phiFa phage infection in the presence of Type III artificial mini-array.**

10-μl aliquots of serial 10x dilutions of phiFa lysate were dropped on lawns formed by *T. thermophilus* HB27c, HB27cΔ*cmr4*, HB27cΔ*csm3*, and HB27cΔ*cmr4*Δ*csm3* strains transformed with a plasmid bearing artificial Type III mini-array targeting phiFa gene 44 (pMK18_Fa_E44) or a control plasmid (pMK18).


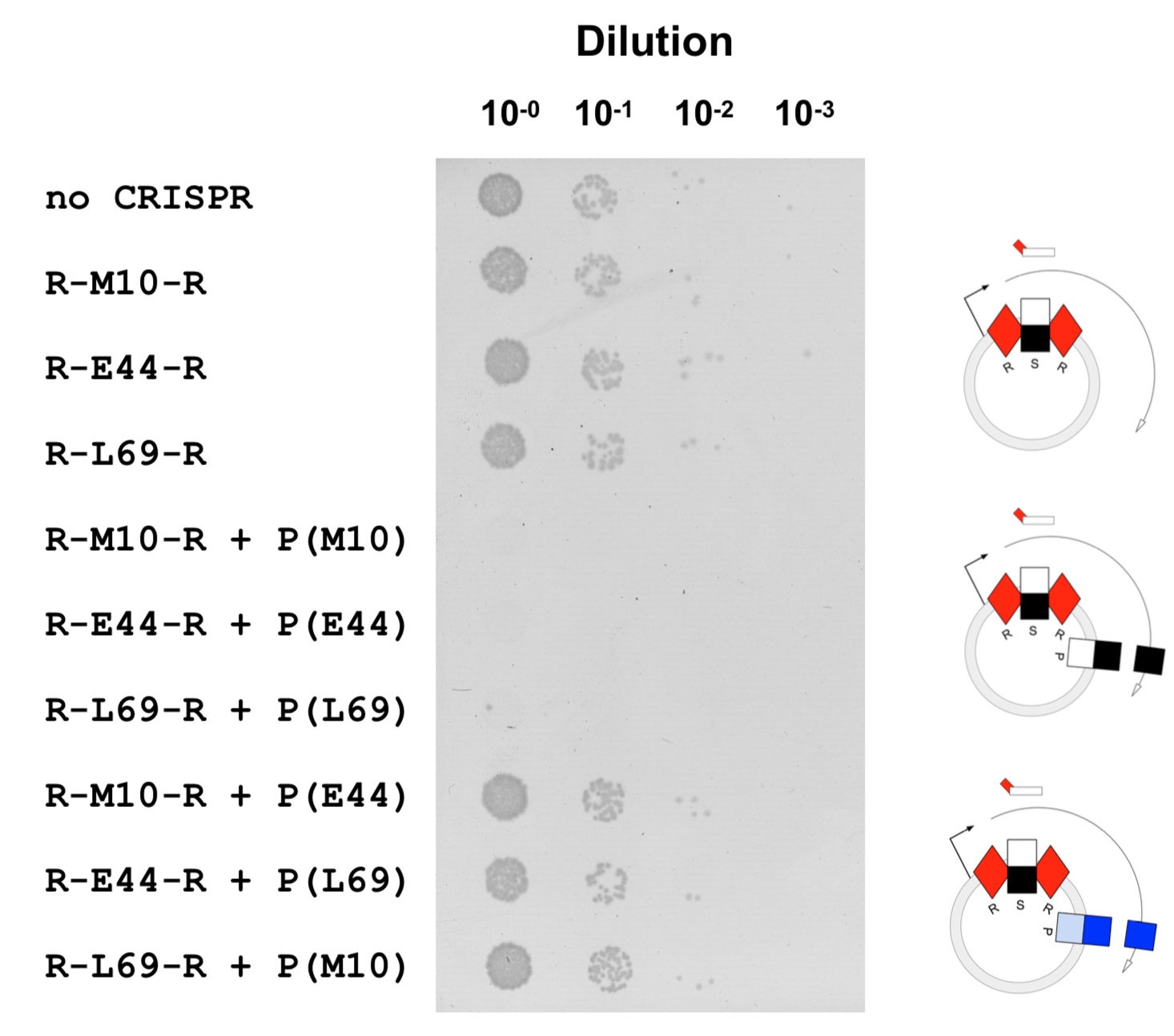


**Supplementary Figure S9. Plasmid-borne Type III spacers corresponding to regions of early, middle, and late phiFa genes are capable to interfere with plasmid transformation.**

*T. thermophilus* HB27c cells were transformed with empty pMK18-based plasmid (“no CRISPR”), control plasmids bearing artificial Type III CRISPR-Cas mini-arrays, or plasmids simultaneously carrying a mini-array and a corresponding protospacer (black) or a non-matching protospacer (blue). Sequences of spacers in mini arrays matched sequences in transcripts of phiFa genes *10* (middle), *69* (late), or *44* (early). 10-fold dilutions of aliquots of transformed cells were plated on selective medium. Lack of growth indicates there is interference.


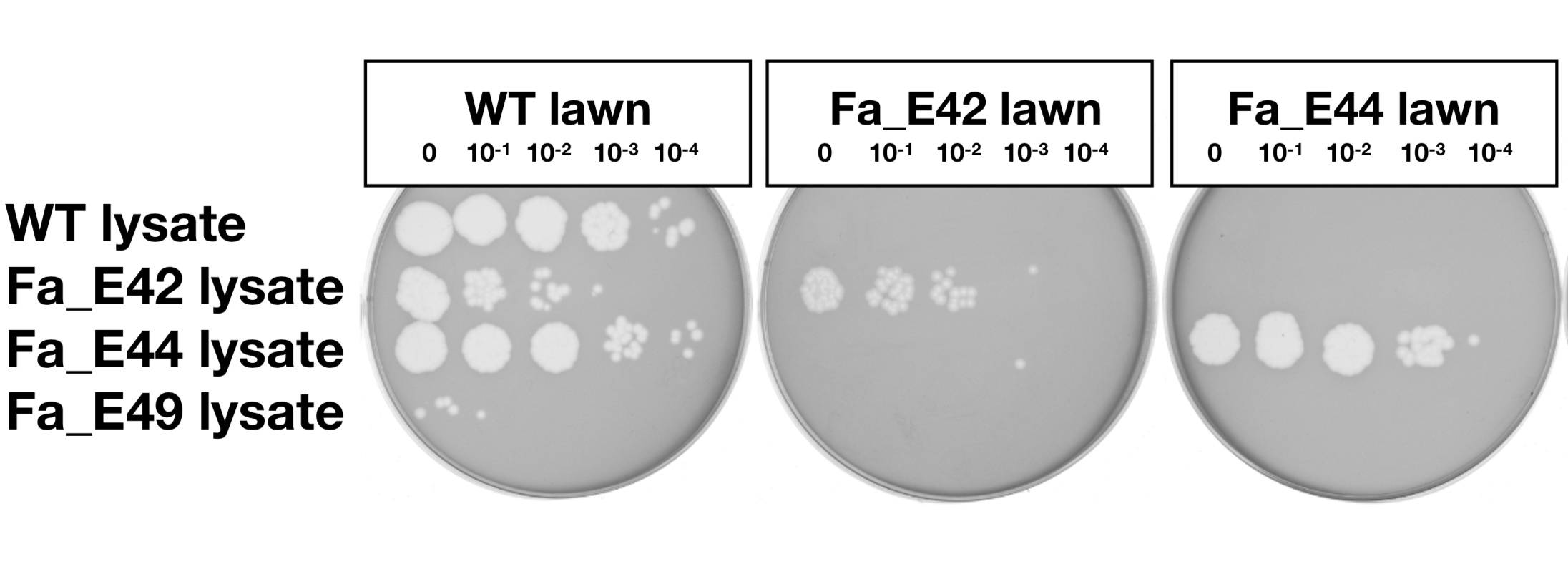


**Supplementary Figure S10. The phiFa phage escapers accumulate during Type III CRISPR-Cas system action.**

Aliquots of cell free supernatants obtained after overnight cultivation in liquid medium of phiFa-infected *T. thermophilus* HB27c cells and cells bearing spacers targeting phiFa genes *42* or *44* transcripts (see Figure 3C) were plated on indicated cell lawns in serial 10x dilutions.


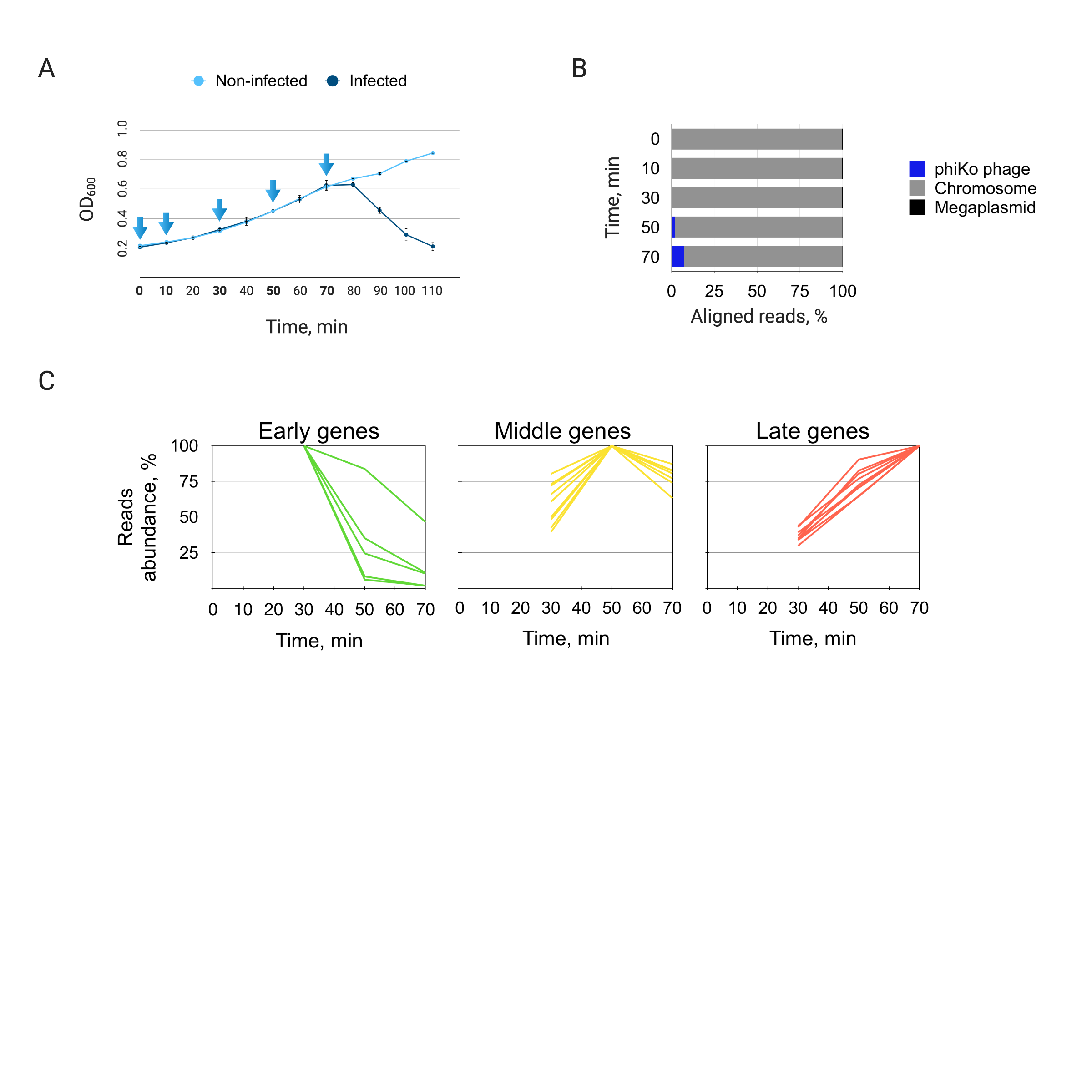


**Supplementary Figure S11. RNA-Seq analysis of *T. thermophilus* HB27c infected with the phiKo phage.**

**(A)** Growth curves for non-infected (light blue) and phiKo infected (dark blue) *T. thermophilus* cultures (MOI=10). The arrows indicate time-points when samples for RNA-Seq analysis were collected. **(B)** Relative quantity of phiKo and *T. thermophilus* HB27c transcripts at different time-points after infection. RNA was purified from cultures at five time points (0, 10, 30, 50, 70 minutes after infection), subjected to RNA-Seq and reads were mapped on the phiKo genome (blue), *T. thermophilus* HB27c chromosome (grey) and megaplasmid (black). **(C)** Time courses of phiKo transcript accumulation. Expression level was normalized to a relative maximum for each particular gene. For each gene the highest expression value was measured. According to the temporal transcript abundances, all genes were grouped into early (curves colored green), middle (curves colored yellow), and late (curves colored red) classes with the maximal expression levels at 30, 50, and 70 minutes after infection, respectively.


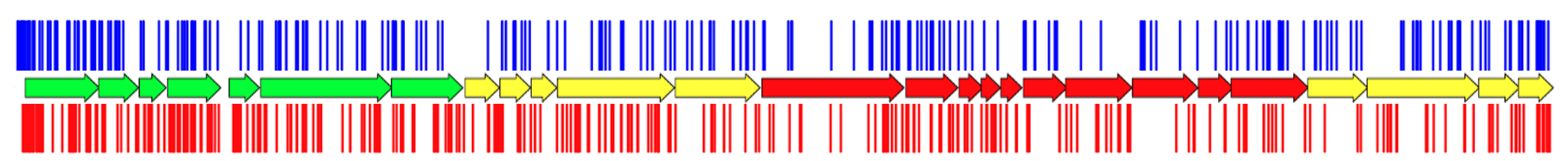


**Supplementary Figure S12. Mapping of unique spacers acquired by CRISPR-2 and CRISPR-11 arrays on the phiKo genome.**

Spacers whose sequences match the “top” strand of phage DNA (5’-3’ direction) are shown as blue bars, those matching the “bottom” strand - as red bars. All spacers presented in CRISPR-2 and CRISPR-11 arrays were combined before mapping. The frequency with which individual spacers were acquired is not considered in this analysis.


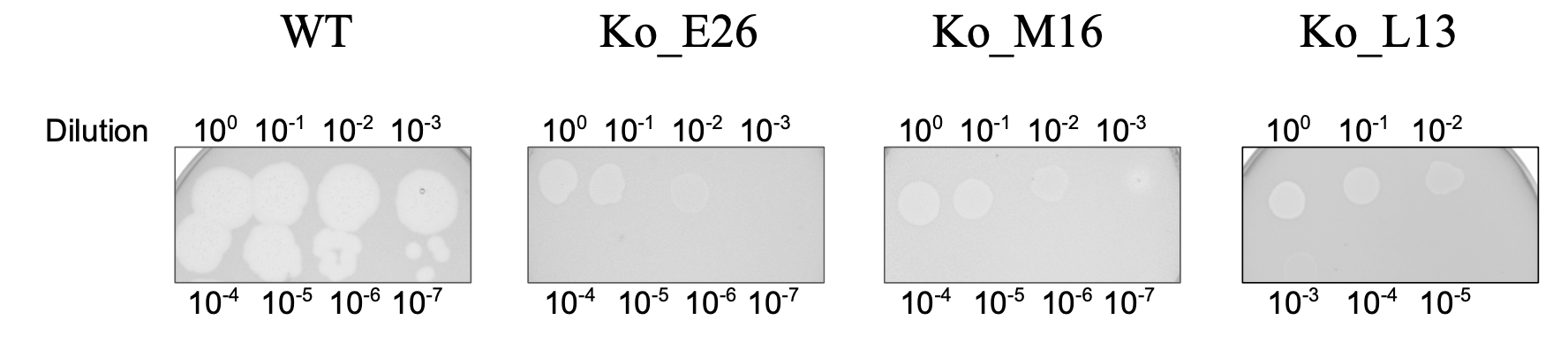


**Supplementary Figure S13. *T. thermophilus* HB27c strains carrying Type III spacers acquired from phiKo genes belonging to different temporal classes are resistant to re-infection.**

10-μl aliquots of serial 10x dilutions of phiKo lysate were dropped on lawns formed by wild-type *T. thermophilus* HB27c or lawns of cells isolated from infected cultures that acquired spacers from the indicated phiKo genes.


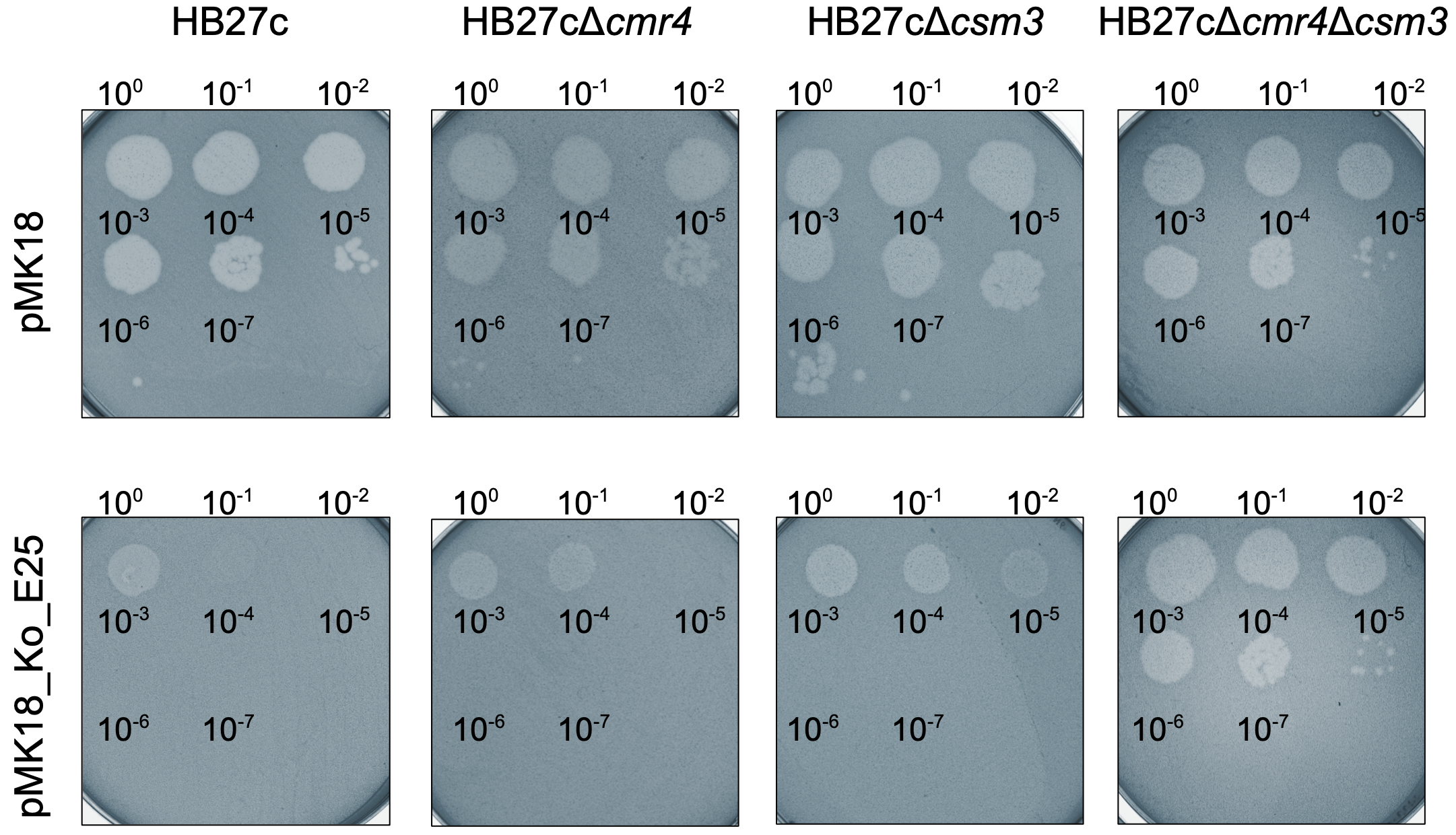


**Supplementary Figure S14. Ability of *T. thermophilus* HB27c WT strain and strains with deactivated III-B (Δ*cmr4*), III-A (Δ*csm3*) or both (Δ*cmr4*Δ*csm3*) interference modules to resist phage infection in the presence of Type III artificial mini-array.**

10-μl aliquots of serial 10x dilutions of phiKo lysate were dropped on lawns formed by *T. thermophilus* HB27c, HB27cΔ*cmr4*, HB27cΔ*csm3*, and HB27cΔ*cmr4*Δ*csm3* strains transformed with a plasmid bearing artificial Type III mini-array targeting phiKo gene 25 (pMK18_Ko_E25) or a control plasmid (pMK18).

**
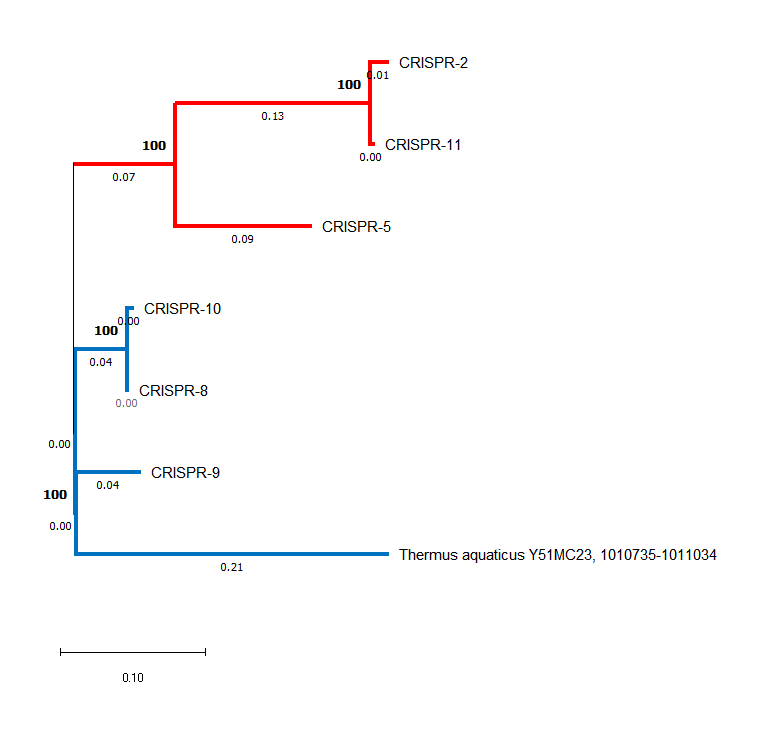
**

**Supplementary Figure S15. Maximum likelihood phylogenetic tree for Type III CRISPR array leader sequences.**

300 nt-long sequences upstream of the first repeat of Type III CRISPR arrays were aligned via Jalview desktop application using TcoffeeWS and a maximum likelihood tree was calculated using phyML. A Type III CRISPR array leader sequence from *Thermus aquaticus* was used as an outgroup. Bootstrap values are shown in bold, length of branches are indicated. The tree is rooted at midpoint.


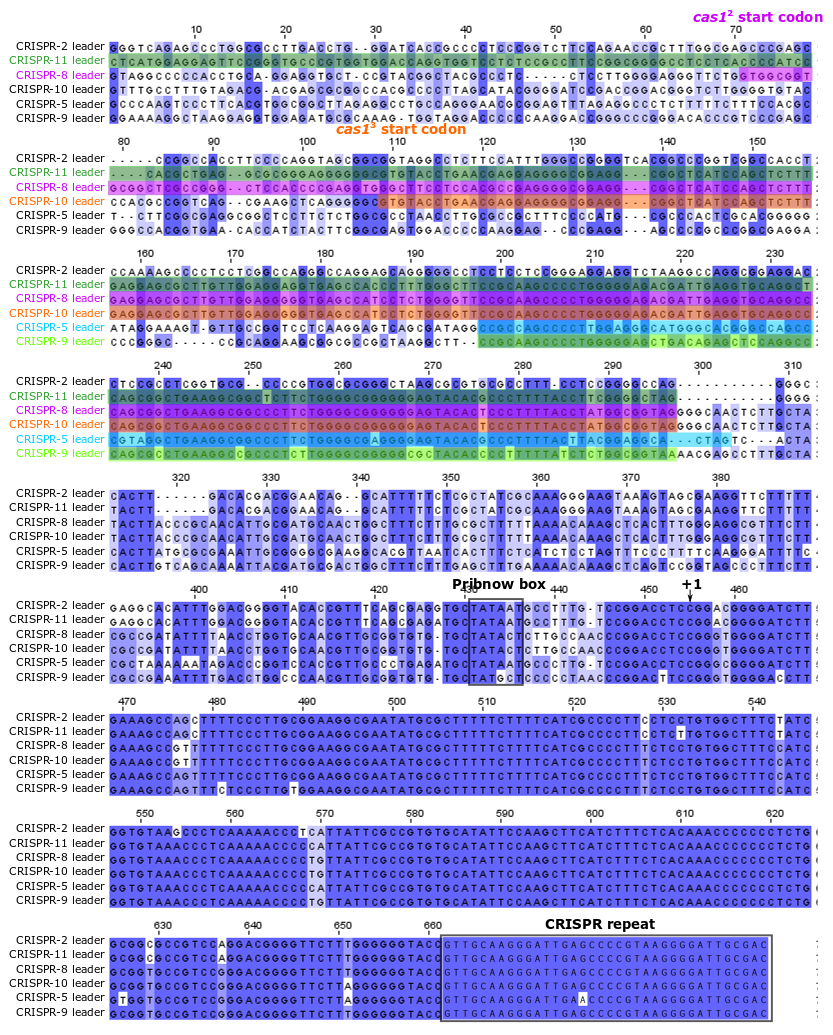


**Supplementary Figure S16. Alignment of Type III CRISPR array leader sequences.**

660 nt-long upstream of the first repeat of *T. thermophilus* HB27c Type III CRISPR arrays were aligned with Jalview. Partial sequences of *cas1* genes are highlighted with background color (green - *cas1*^4^ gene upstream of the CRISPR-11 leader; purple - *cas1*^2^ gene upstream of the CRISPR-8 leader; orange - *cas1*^3^ gene upstream of the CRISPR-10 leader; light blue – a *cas1* gene fragment upstream of the CRISPR-5 leader; lime – a *cas1* gene fragment upstream of the CRISPR-9 leader. Possible start codons for short *cas1*^2^ and *cas1*^3^ genes are indicated. Transcription start sites predicted from RNAseq data and likely Pribnow boxes of CRISPR array promoters are also indicated.

**Supplementary tables**

**Supplementary Table S1. Oligonucleotides used in the study.**

| Name | Sequence 5′ to 3′ |
| --- | --- |
| Detection of adaptation | |
| CRISPR-1-F | CGCCATGTTGAGATGATAAGGG |
| CRISPR-2,5,8,9,10,11-F | TGTGCATATTCCAAGCTTCATCT |
| CRISPR-3,4-F | GCGAAGGACCTCCTTCCTGC |
| CRISPR-6,7-F | GCGTGCGAAAATGGCCTCAAGAC |
| CRISPR-1-R | GCCCTGCATCGTCATCGCG |
| CRISPR-2-R | GGGATAAAGAGATATACCCCA |
| CRISPR-3-R | GCCTTCTCACCTATGACCTC |
| CRISPR-4-R | ATGACCTGCTATGCCCCGTG |
| CRISPR-5-R | CTCACCTAGGAACCTCGTCAG |
| CRISPR-6-R | TCAGTATACCACAGCTCTGC |
| CRISPR-7-R | TCGCCGTTCACGAGCCGGA |
| CRISPR-8,9-R | GTTTGCGTGGATCCTGAAAGAG |
| CRISPR-10-R | CTCGCACCGTCATCCTTG |
| CRISPR-11-R | GCAACTAGCGGTCCACCC |
| Construction of HB27c derivatives lacking *cas1^1/4^* | |
| Hyg-F | GCACAGATGGTCATAACCTGAAGGA |
| Hyg-R | CGGGCCGGGGATCGATC |
| Bleo-F | TTAGTCCTGCTCCTCGGCCAC |
| Bleo-R | CCCCGGGAGTATAACAGAAACCTTAAG |
| dCas1-1_L(pT7)-F | atatcggatccccgggtaccAAGAGCAGTCTTCTGTACAGCAA |
| dCas1-1_L(Hyg)-R | caggttatgaccatctgtgcGGGAACTGTACCTGGTCATCG |
| dCas1-1_R(Hyg)-F | ggggatcgatccccggcccgGCTTCGTTTTCCAGGACGTAG |
| dCas1-1_R(pT7)-R | cggccagtgaattcgagctcGGCCAAGGAAGAGGCCTAC |
| dCas1-4_L_pT7-F | gatctactagtcatatggatCAGGAGAGGCTGGGCTTCTC |
| dCas1-4_L_Hyg-R | caggttatgaccatctgtgcGCCTAGAACCTGGGCCAG |
| dCas1-4_R_Hyg-F | tctgttatactccccccgggACCAGGTGGTCCTCTCCG |
| dCas1-4_R_pT7-R | tcggtacccggggatccgatCCCTAGCCCCGAAGGTAAAAGG |
| dCas1-4_L_Bleo-R | gtggccgaggagcaggactaaGCCTAGAACCTGGGCCAG |
| dCas1-4_R_Bleo-F | tttctgttatactcccggggACCAGGTGGTCCTCTCCG |
| Construction of plasmids bearing CRISPR mini-arrays | |
| Fa_ORF3-F | *AGCTT*GTTGCAAGGGATTGAGCCCCGTAAGGGGATTGCGAC**CACGTAGGAGTAGTAAAGCGCCGACAGGTCCAGCCCGA**GTTGCAAGGGATTGAGCCCCGTAAGGGGATTGCGAC*G* |
| Fa_ORF3-R | *TCGAC*GTCGCAATCCCCTTACGGGGCTCAATCCCTTGCAAC**TCGGGCTGGACCTGTCGGCGCTTTACTACTCCTACGTG**GTCGCAATCCCCTTACGGGGCTCAATCCCTTGCAAC*A* |
| Fa_ORF10-F | *AGCTT*GTTGCAAGGGATTGAGCCCCGTAAGGGGATTGCGAC**TCACACGCCGCTTCCGCGGCCCATGCGCACGGAAGGC**GTTGCAAGGGATTGAGCCCCGTAAGGGGATTGCGAC*G* |
| Fa_ORF10-R | *TCGAC*GTCGCAATCCCCTTACGGGGCTCAATCCCTTGCAAC**GCCTTCCGTGCGCATGGGCCGCGGAAGCGGCGTGTGA**GTCGCAATCCCCTTACGGGGCTCAATCCCTTGCAAC*A* |
| Fa_ORF19-F | *AGCTT*GTTGCAAGGGATTGAGCCCCGTAAGGGGATTGCGAC**CACACCTCCTTCTCTTCGCTGTGCAGCGGAGCTAC**GTTGCAAGGGATTGAGCCCCGTAAGGGGATTGCGAC*G* |
| Fa_ORF19-R | *TCGAC*GTCGCAATCCCCTTACGGGGCTCAATCCCTTGCAAC**GTAGCTCCGCTGCACAGCGAAGAGAAGGAGGTGTG**GTCGCAATCCCCTTACGGGGCTCAATCCCTTGCAAC*A* |
| Fa_ORF25-F | *AGCTT*GTTGCAAGGGATTGAGCCCCGTAAGGGGATTGCGAC**TCGTGAGGGAGGAAGAAGTTTGGGTAACGGCGGATAA**GTTGCAAGGGATTGAGCCCCGTAAGGGGATTGCGAC*G* |
| Fa_ORF25-R | *TCGAC*GTCGCAATCCCCTTACGGGGCTCAATCCCTTGCAAC**TTATCCGCCGTTACCCAAACTTCTTCCTCCCTCACGA**GTCGCAATCCCCTTACGGGGCTCAATCCCTTGCAAC*A* |
| Fa_ORF26-F | *AGCTT*GTTGCAAGGGATTGAGCCCCGTAAGGGGATTGCGAC**CTGTTGTATACGAACAGTACGTTGTCAACGTGAGT**GTTGCAAGGGATTGAGCCCCGTAAGGGGATTGCGAC*G* |
| Fa_ORF26-R | *TCGAC*GTCGCAATCCCCTTACGGGGCTCAATCCCTTGCAAC**ACTCACGTTGACAACGTACTGTTCGTATACAACAG**GTCGCAATCCCCTTACGGGGCTCAATCCCTTGCAAC*A* |
| Fa_ORF32-F | *AGCTT*GTTGCAAGGGATTGAGCCCCGTAAGGGGATTGCGAC**CAATCTTCTTCAGCCGGTGCCTGCACTCGGGGTTG**GTTGCAAGGGATTGAGCCCCGTAAGGGGATTGCGAC*G* |
| Fa_ORF32-R | *TCGAC*GTCGCAATCCCCTTACGGGGCTCAATCCCTTGCAAC**CAACCCCGAGTGCAGGCACCGGCTGAAGAAGATTG**GTCGCAATCCCCTTACGGGGCTCAATCCCTTGCAAC*A* |
| Fa_ORF33-F | *AGCTT*GTTGCAAGGGATTGAGCCCCGTAAGGGGATTGCGAC**CTCGTCGGTGAGTTCCACCTCGTTGGCGATGCGTTCTAG**GTTGCAAGGGATTGAGCCCCGTAAGGGGATTGCGAC*G* |
| Fa_ORF33-R | *TCGAC*GTCGCAATCCCCTTACGGGGCTCAATCCCTTGCAAC**CTAGAACGCATCGCCAACGAGGTGGAACTCACCGACGAG**GTCGCAATCCCCTTACGGGGCTCAATCCCTTGCAAC*A* |
| Fa_ORF36-F | *AGCTT*GTTGCAAGGGATTGAGCCCCGTAAGGGGATTGCGAC**TCGCGAAACTTCAGGTGTCTAGCTTTTCTTGTAGGCCGG**GTTGCAAGGGATTGAGCCCCGTAAGGGGATTGCGAC*G* |
| Fa_ORF36-R | *TCGAC*GTCGCAATCCCCTTACGGGGCTCAATCCCTTGCAAC**CCGGCCTACAAGAAAAGCTAGACACCTGAAGTTTCGCGA**GTCGCAATCCCCTTACGGGGCTCAATCCCTTGCAAC*A* |
| Fa_ORF44-F | *AGCTT*GTTGCAAGGGATTGAGCCCCGTAAGGGGATTGCGAC**ACTGACCTTCTTCGTCGATTCGACCGGTGTACACCGG**GTTGCAAGGGATTGAGCCCCGTAAGGGGATTGCGAC*G* |
| Fa_ORF44-R | *TCGAC*GTCGCAATCCCCTTACGGGGCTCAATCCCTTGCAAC**CCGGTGTACACCGGTCGAATCGACGAAGAAGGTCAGT**GTCGCAATCCCCTTACGGGGCTCAATCCCTTGCAAC*A* |
| Fa_ORF44^REV^-F | *AGCTT*GTTGCAAGGGATTGAGCCCCGTAAGGGGATTGCGAC**CCGGTGTACACCGGTCGAATCGACGAAGAAGGTCAGT**GTTGCAAGGGATTGAGCCCCGTAAGGGGATTGCGAC*G* |
| Fa_ORF44^REV^-R | *TCGAC*GTCGCAATCCCCTTACGGGGCTCAATCCCTTGCAAC**ACTGACCTTCTTCGTCGATTCGACCGGTGTACACCGG**GTCGCAATCCCCTTACGGGGCTCAATCCCTTGCAAC*A* |
| Fa_ORF47-F | *AGCTT*GTTGCAAGGGATTGAGCCCCGTAAGGGGATTGCGAC**TTTGCCAGGTACCCGCTACCGATACGCTTGACCTTAAT**GTTGCAAGGGATTGAGCCCCGTAAGGGGATTGCGAC*G* |
| Fa_ORF47-R | *TCGAC*GTCGCAATCCCCTTACGGGGCTCAATCCCTTGCAAC**ATTAAGGTCAAGCGTATCGGTAGCGGGTACCTGGCAAA**GTCGCAATCCCCTTACGGGGCTCAATCCCTTGCAAC*A* |
| Fa_ORF49-F | *AGCTT*GTTGCAAGGGATTGAGCCCCGTAAGGGGATTGCGAC**GGCGGTGACTTTGACTCCACCACCACCGCGCTCAACTAAG**GTTGCAAGGGATTGAGCCCCGTAAGGGGATTGCGAC*G* |
| Fa_ORF49-R | *TCGAC*GTCGCAATCCCCTTACGGGGCTCAATCCCTTGCAAC**CTTAGTTGAGCGCGGTGGTGGTGGAGTCAAAGTCACCGCC**GTCGCAATCCCCTTACGGGGCTCAATCCCTTGCAAC*A* |
| Fa_ORF55-F | *AGCTT*GTTGCAAGGGATTGAGCCCCGTAAGGGGATTGCGAC**CGGTGTAAATCTCCAGCTGCCAGCCTTCAGGCAAAG**GTTGCAAGGGATTGAGCCCCGTAAGGGGATTGCGAC*G* |
| Fa_ORF55-R | *TCGAC*GTCGCAATCCCCTTACGGGGCTCAATCCCTTGCAAC**CTTTGCCTGAAGGCTGGCAGCTGGAGATTTACACCG**GTCGCAATCCCCTTACGGGGCTCAATCCCTTGCAAC*A* |
| Fa_ORF69-F | *AGCTT*GTTGCAAGGGATTGAGCCCCGTAAGGGGATTGCGAC**TAGTTGTATGAAGAGCCTGGGTCAATCACAACGATGTCGC**GTTGCAAGGGATTGAGCCCCGTAAGGGGATTGCGAC*G* |
| Fa_ORF69-R | *TCGAC*GTCGCAATCCCCTTACGGGGCTCAATCCCTTGCAAC**GCGACATCGTTGTGATTGACCCAGGCTCTTCATACAACTA**GTCGCAATCCCCTTACGGGGCTCAATCCCTTGCAAC*A* |
| Ko_ORF25-F | AGCTTGTTGCAAGGGATTGAGCCCCGTAAGGGGATTGCGACGCTCTGGATCTCCACCAGGACGCTCACCGCCTTGAGCCGGTTGCAAGGGATTGAGCCCCGTAAGGGGATTGCGACA |
| Ko_ORF25-R | AGCTTGTCGCAATCCCCTTACGGGGCTCAATCCCTTGCAACCGGCTCAAGGCGGTGAGCGTCCTGGTGGAGATCCAGAGCGTCGCAATCCCCTTACGGGGCTCAATCCCTTGCAACA |
| Construction of plasmids bearing artificial Type III CRISPR-Cas mini-arrays and corresponding protospacers | |
| Proto_PvuI_ORF10-F | *CG***TCACACGCCGCTTCCGCGGCCCATGCGCACGGAAGGC***CGAT* |
| Proto_PvuI_ORF10-R | *CG***GCCTTCCGTGCGCATGGGCCGCGGAAGCGGCGTGTGA***CGAT* |
| Proto_PvuI_ORF44-F | *CG***ACTGACCTTCTTCGTCGATTCGACCGGTGTACACCGG***CGAT* |
| Proto_PvuI_ORF44-R | *CG***CCGGTGTACACCGGTCGAATCGACGAAGAAGGTCAGT***CGAT* |
| Proto_PvuI_ORF69-F | *CG***TAGTTGTATGAAGAGCCTGGGTCAATCACAACGATGTCGC***CGAT* |
| Proto_PvuI_ORF69-R | *CG***GCGACATCGTTGTGATTGACCCAGGCTCTTCATACAACTA***C*G*AT* |
| Analysis of escaper diversity | |
| 42_HTS-F | GTGAATGTTAGCAGGTTGACGG |
| 42_HTS-R | CGCCGTATACGAATAGTCAGGG |
| 44_HTS-F | GCCGGTACTACCAGGACTTTG |
| 44_HTS-R | GAAGAGAAGACGGCGGAGTTG​ |

**Supplementary Table S2. Sequences of *T. thermophilus* HB27c CRISPR repeats.**

| **Sequence of repeat** | **length, bp** | **CRISPR subtype** |
| --- | --- | --- |
| **GTTGCAAGGGATTGARCCCCGTAAGGGGATTGCGAC** | 36 | III-A and III-B |
| **GTTGCACCGGCCCGAAAGGGCCGGTGAGGATTGAAAC** | 37 | I-C |
| **GTTGCAAACCTCGTTAGCCTCGTAGAGGATTGAAAC** | 36 | I-B |

**Supplementary Table S3. CRISPR-associated genes of *T. thermophilus* HB27c.**

| **gene** | **ORF** | **start** | **end** |
| --- | --- | --- | --- |
| **Megaplasmid** | | | |
| III-A/B | | | |
| *CARF^3^* | HB27c_P00142 | 124785 | 125990 |
| *cas1^4^* | HB27c_P00143 | 125994 | 126941 |
|  | | | |
| *cas2^1^* | HB27c_P00146 | 130082 | 130354 |
| III-A | | | |
| *csm1* | HB27c_P00147 | 130410 | 132827 |
| *csm2* | HB27c_P00148 | 132828 | 133232 |
| *csm3* | HB27c_P00149 | 133244 | 133969 |
| *csm4* | HB27c_P00150 | 133979 | 134854 |
| *csm5* | HB27c_P00151 | 134851 | 136005 |
| III-A/B | | | |
| *CARF^1^* | HB27c_P00152 | 136016 | 137410 |
|  | | | |
| *CARF^2^* | HB27c_P00154 | 139208 | 141118 |
|  | | | |
| III-B | | | |
| *cmr2* | HB27c_P00159 | 143798 | 145552 |
| *cmr3* | HB27c_P00160 | 145542 | 146627 |
| *cmr1* | HB27c_P00161 | 146624 | 147811 |
| *cmr4* | HB27c_P00162 | 147827 | 148687 |
| *cmr5* | HB27c_P00163 | 148691 | 149035 |
| *cmr6* | HB27c_P00164 | 149038 | 150084 |
|  | | | |
| I-C | | | |
| *cas3* | HB27c_P00176 | 169966 | 172149 |
| *cas5* | HB27c_P00177 | 172159 | 172848 |
| *cas8* | HB27c_P00178 | 172832 | 174595 |
| *cas7* | HB27c_P00179 | 174595 | 175746 |
| *cas4^1^* | HB27c_P00180 | 175736 | 176086 |
|  | | | |
| I-B | | | |
| *cas2^2^* | HB27c_P00243 | 230148 | 230432 |
| *cas1^1^* | HB27c_P00244 | 230429 | 231406 |
| *cas4^2^* | HB27c_P00245 | 231397 | 231981 |
| *WYL* | HB27c_P00246 | 232325 | 233329 |
| *cas8* | HB27c_P00247 | 233347 | 235647 |
| *cas7* | HB27c_P00248 | 235649 | 236530 |
| *cas5* | HB27c_P00249 | 236532 | 237323 |
| *cas3`* | HB27c_P00250 | 237290 | 239743 |
| *cas6^1^* | HB27c_P00251 | 239743 | 240537 |
|  | | | |
| Type III-A/B | | | |
| *cas1^2^* | HB27c_P00263 | 251509 | 251288 |
|  | | | |
| **Chromosome** | | | |
| Type III-A/B | | | |
| *cas1^3^* | HB27c_C01208 | 1154430 | 1154245 |
|  | | | |
| Type III-A/B | | | |
| *cas2^3^* | HB27c_C01992 | 1848785 | 1849021 |
| *cas6^2^* | HB27c_C01993 | 1849024 | 1849743 |

**Table S4. Spacers acquired during infection of *T. thermophilus* HB27c with phiFa or phiKo in cells that survived the infection.**

|  | spacer #1 | | | | | spacer #2 | | | | |
| --- | --- | --- | --- | --- | --- | --- | --- | --- | --- | --- |
| strain | sequence of spacer (5’-3’) | # of CRISPR array | length | ORF | orientation | sequence of spacer (5’-3’) | # of CRISPR array | length | ORF | orientation |
| Fa_E42 | CTTTTGAAGCCAACCGTGAAGAAGCTTAGCGTTGTTCAG | 11 | 39 bp | phiFa_42 | anti  parallel | - | - | - | - | - |
| Fa_E44 | ACTGACCTTCTTCGTCGATTCGACCGGTGTACACCGG | 2 | 37 bp | phiFa_44 | anti  parallel | - | - | - | - | - |
| Fa_E44.2 | CACCGGCCTAACAGCCAACTCCGCCGTCTTCTCTTCAATCA | 2 | 41bp | phiFa_44 | anti  parallel | - | - | - | - | - |
| Fa_E49 | GTTGACCAGCTCATCGGCATCGGTGCCGAAGAAGGCAT | 2 | 38 bp | phiFa_49 | anti  parallel | - | - | - | - | - |
| Fa_E42_Fa_E43 | CTTTTGAAGCCAACCGTGAAGAAGCTTAGCGTTGTTCAG | 11 | 39 bp | phiFa_42 | anti  parallel | TTGTTCAAGAGCGCGACATCGGTCTCCTGAATGGGCACAT | 2 | 40 bp | phiFa_43 | anti  parallel |
| Fa_E42_HB27с | CTTTTGAAGCCAACCGTGAAGAAGCTTAGCGTTGTTCAG | 11 | 39 bp | phiFa_42 | anti  parallel | CCGACCTCCTTGTCCTCCCCGGGCAGGGCCACTTC | 2 | 35 bp | chr  _0062 | parallel |
| HB27c.2_  Fa_E44.3 | CCGCCAAGAACCTCTCCTTCAGCGCCTTTGAGCCCTCG | 11 | 38 bp | chr  _1129 | parallel | ATGCAGAGAAGTCGGAGTCCACTGACCTTCTTCGTCGA | 11 | 38 bp | phiFa_44 | anti  parallel |
| Ko_E26 | CCCACTTCCCCCGACTCAAGCCCGAGCGCCCCGCCAACCACGA | 2 | 43 bp | phiKo_26 | anti  parallel | - | - | - | - | - |
| Ko_E26.2 | GCGAGCACCGTGCGAGCCGCCGCGCCAATAAGATGGG | 2 | 37 bp | phiKo_26 | anti  parallel | - | - | - | - | - |
| Ko_E26.3 | TTCCGCCAAAGAGTAGTATCCACGCCAAGACATCTTCGG | 11 | 39 bp | phiKo_26 | anti  parallel | - | - | - | - | - |
| Ko_E20 | TTCCATCCGGGGGCCCGCTCGTTCCACTCAGGAGGGTAGC | 2 | 40 bp | phiKo_20 | anti  parallel | - | - | - | - | - |
| Ko_M16 | CACCATTACCGCCGCTATCTCCCGAGCCTGCGCCTCCGG | 2 | 39 bp | phiKo_16 | anti  parallel | - | - | - | - | - |
| Ko_M16.2 | TTGAGCTTTAGCCCAATCCTCCAGGGCCTTGCCCCACG | 2 | 38 bp | phiKo_16 | anti  parallel | - | - | - | - | - |
| Ko_L13 | AGCTCCACCCTCCAACGGAGGGGAGAACGTTATTGAG | 11 | 37 bp | phiKo_13 | anti  parallel | - | - | - | - | - |

**Supplementary Table S5. Point mutations identified by whole genome sequencing of four phiFa escapers.**

| position | REF* | ALT** | ORF42 isolate#1 | ORF42 isolate#2 | ORF44  isolate  #1 | ORF44 isolate  #2 | changes in amino acids | gene |
| --- | --- | --- | --- | --- | --- | --- | --- | --- |
| 5326 | G | A |  | 1 |  |  | Leu -> Phe | ORF05 (ribonucleoside-triphosphate reductase) |
| 7055 | C | T |  | 1 |  |  | Ala -> Thr | ORF08 (hypothetical protein) |
| 12502 | G | A |  |  | 1 |  |  | intergenic |
| 28991 | C | A | 1 |  |  |  | Lys -> Asn | ORF42 (hypothetical protein) |
| 61851 | T | C | 1 | 1 |  |  | Leu -> Pro | ORF70 (hypothetical protein) |

* - reference, ** - alternative

**Supplementary Table S6. Transcription of CRISPR arrays and *cas1* genes.**

|  | **location** | **CRISPR type** | **number of spacers** | **transcription level of CRISPR array (RPKM)** | **type III leader sequence** | **size of Сas1 upstream** | **distance to *cas1* upstream** | **transcription level of *cas1* (RPKM)** |
| --- | --- | --- | --- | --- | --- | --- | --- | --- |
| **CRISPR-1** | megaplasmid | III | 3 | 2,57 | - | - | - | - |
| **CRISPR-2** | megaplasmid | III | 14 | 39,96 | + | - | - | - |
| **CRISPR-5** | megaplasmid | III | 6 | 62,00 | + | 34 aa | 357 bp | - |
| **CRISPR-8** | megaplasmid | III | 9 | 72,29 | + | 87 aa (Сas1^2^) | 363 bp | 4,13 |
| **CRISPR-9** | chromosome | III | 2 | 266,19 | + | 35 aa | 366 bp | - |
| **CRISPR-10** | chromosome | III | 6 | 84,01 | + | 74 aa (Сas1^3^) | 363 bp | 5,58 |
| **CRISPR-11** | megaplasmid | III | 18 | 56,32 | + | 315 aa (Сas1^4^) | 344 bp | 3,64 |
| **CRISPR-3** | megaplasmid | I-C | 7 | 56,45 | N/A | - | - | - |
| **CRISPR-4** | megaplasmid | I-C | 7 | 100,93 | N/A | - | - | - |
| **CRISPR-6** | megaplasmid | I-B | 6 | 60,86 | N/A | 325 aa (Сas1^1^) | - | 0,58 |
| **CRISPR-7** | megaplasmid | I-B | 13 | 20,04 | N/A | - | - | - |

**Supplementary methods**

**Interference assays with *T. thermophilus* HB27c derivative strains bearing deactivated III-A and III-B CRISPR-Cas systems.**

*T. thermophilus* strains with deactivated III-B (Δ*cmr4*) or both III-A and III-B (Δ*cmr4*Δ*csm3*) interference modules were constructed in our lab previously (1). A derivative with an inactive III-A system was made by replacement of the *csm3* gene in wild-type *T. thermophilus* HB27c strain with a thermostable hygromycin resistance marker via natural homologous recombination using the protocol described for construction of ∆*cas1^1^*, ∆*cas1^4^*, and ∆*cas1^1^*∆*cas1^4^* strains (see chapter “Methods”). Complementary oligonucleotides Ko_ORF25-F and Ko_ORF25-R (see Supplementary Table S1) were annealed and cloned into the HindIII site of pMK18-based plasmid with a transcription terminator (1). *T. thermophilus* HB27c WT, Δ*cmr4*, Δ*csm3* and Δ*cmr4*Δ*csm3* cells were transformed with the resulting plasmid. 10-μl aliquots of serial 10x dilutions of phiKo lysate were dropped on lawns formed by each of the strains bearing the plasmid with Type III artificial mini-array targeting phiKo gene 25.

**Analysis of CRISPR-Cas systems transcription**

Control samples of *Thermus* culture before phiKo infection were used for analysis of CRISPR-Cas systems transcription. RNA sequencing was carried out at Illumina platform using the resources of the Skoltech Genomics Core Facility. Reads were mapped on the bacterial genome with bowtie2 program (2). Total read coverage for each CRISPR array (count table) was calculated with multicov function from bedtools utilities (3). Count table was transformed into RPKM values using edgeR package (4). Mean calculated for two biological replicas is presented.

**Supplementary references**

1. You,L., Ma,J., Wang,J., Artamonova,D., Wang,M., Liu,L., Xiang,H., Severinov,K., Zhang,X. and Wang,Y. (2019) Structure Studies of the CRISPR-Csm Complex Reveal Mechanism of Co-transcriptional Interference. *Cell*, 176, 239-253.e16.
2. Langmead B. and Salzberg S.L. (2012) Fast gapped-read alignment with Bowtie 2. *Nat. Methods*, 9, 357–359.
3. Quinlan A.R. (2014) BEDTools: The Swiss-Army tool for genome feature analysis. *Curr. Protoc. Bioinformatics*, 47, 11.12.1-11.12.34.
4. Robinson M.D., McCarthy D.J., Smyth G.K. (2010) edgeR: a Bioconductor package for differential expression analysis of digital gene expression data, *Bioinformatics*, 26(1), 139-140.
